# Comprehensive Maps of *Drosophila* Higher Olfactory Centers: Spatially Segregated Fruit and Pheromone Representation

**DOI:** 10.1016/j.cell.2007.01.040

**Published:** 2007-03-23

**Authors:** Gregory S.X.E. Jefferis, Christopher J. Potter, Alexander M. Chan, Elizabeth C. Marin, Torsten Rohlfing, Calvin R. Maurer, Liqun Luo

**Affiliations:** 1 Department of Biological Sciences, Stanford University, Stanford, CA 94305, USA; 2 Neurosciences Program, Stanford University, Stanford, CA 94305, USA; 3 Department of Neurosurgery, Stanford University, Stanford, CA 94305, USA; 4 Howard Hughes Medical Institute, Stanford University, Stanford, CA 94305, USA; 5 Department of Zoology, University of Cambridge, Downing Street, Cambridge, CB2 3EJ, UK; 6 Neuroscience Program, SRI International, Menlo Park, CA 94025, USA

**Keywords:** SYSNEURO

## Abstract

In *Drosophila*, ∼50 classes of olfactory receptor neurons (ORNs) send axons to 50 corresponding glomeruli in the antennal lobe. Uniglomerular projection neurons (PNs) relay olfactory information to the mushroom body (MB) and lateral horn (LH). Here, we combine single-cell labeling and image registration to create high-resolution, quantitative maps of the MB and LH for 35 input PN channels and several groups of LH neurons. We find (1) PN inputs to the MB are stereotyped as previously shown for the LH; (2) PN partners of ORNs from different sensillar groups are clustered in the LH; (3) fruit odors are represented mostly in the posterior-dorsal LH, whereas candidate pheromone-responsive PNs project to the anterior-ventral LH; (4) dendrites of single LH neurons each overlap with specific subsets of PN axons. Our results suggest that the LH is organized according to biological values of olfactory input.

## Introduction

**Figure 1:**
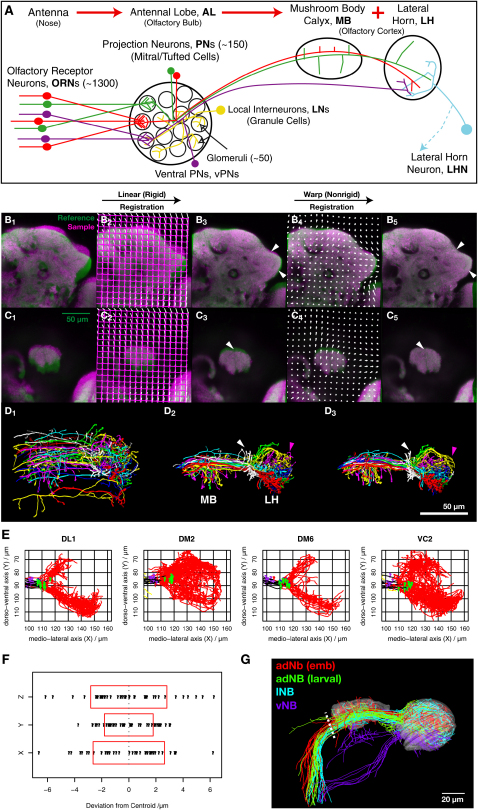
Nonrigid Registration and Accuracy Estimation (A) Organization of the *Drosophila* olfactory system (vertebrate counterparts in parentheses). Each color represents ORNs expressing a given olfactory receptor or their postsynaptic PN partners. Most PN axons pass through the MB calyx, forming en passant synapses, before terminating in the LH. A small group (vPNs) project directly to the LH. (B and C) Example of registration, showing an optical section at the level of the LH (B) and the MB calyx (C). Initially the sample (magenta) and reference (green) brains are unregistered (B_1_ and C_1_). The first registration process calculates a linear (rigid) registration (B_2_ and C_2_). The result (B_3_ and C_3_) is the starting point for warping (nonrigid) registration that allows smoothly varying deformations across the whole brain. (B_4_) and (C_4_) show the resultant deformation vector field (arrows indicate the direction of movement from rigid to nonrigid coordinates). The final stage is in almost perfect alignment (B_5_ and C_5_). Arrowheads indicate regions showing significant improvement after warping registration. Grids in (B_2_), (B_4_), (C_2_), and (C_4_) were at 10μm spacing before registration. (D) Application of calculated deformation fields to neuronal tracings. 35 PNs consisting of 5 examples from 7 classes before (D_1_), after rigid (D_2_), and full nonrigid (D_3_) registration. The transition following the initial rigid registration appears more dramatic, but neurons within the same class are more tightly colocalized after the nonrigid registration: compare axonal processes of PNs in white and magenta (arrowheads). PN glomerular classes: 1, white; DA1, red; DM5, yellow; DM6, green; VA1d, cyan; VA1lm, blue; VM2, magenta. See Movies S1 and S2 for an animation. (E) Location of identified branch points (green dot) for 4 PN classes. Axons are colored black before and red after reaching the LH. These branch points are used to estimate registration accuracy in (F). (F) Distance of the branch point in each PN from the mean position (centroid) of all the branch points of that PN class. Dotted line shows mean position and red box ± one standard deviation. (G) PNs of different developmental origins have segregated axons in the inner antennocerebral tract. Red, green, cyan and purple represent axons from embryonically born PNs of the anterodorsal neuroblast and larval born PNs of the anterodorsal, lateral and ventral neuroblasts, respectively.

The first olfactory relay in the brain contains a spatial map. Olfactory receptor neurons (ORNs) expressing a specific odorant receptor (and therefore having precisely defined olfactory tuning properties) send axon projections to discrete and reproducibly positioned glomeruli in the vertebrate olfactory bulb or insect antennal lobe (Figure 1A; Bargmann, 2006). In *Drosophila*, most ORN classes express one specific odorant receptor and send axons to one of ∼50 glomerular targets (Laissue et al., 1999; Couto et al., 2005; Fishilevich and Vosshall, 2005).

Persistent spatial organization deep within the brain is a motif in many sensory systems. For example, adjacent regions of the somatosensory cortex respond to stimuli from neighboring body parts (Penfield and Rasmussen, 1950). Does the spatial organization evident in the first olfactory relay also persist at deeper levels? In flies, Marin et al. (2002) and Wong et al. (2002) have described the branching patterns of the axons of second order projection neurons (PNs, equivalent to vertebrate mitral cells) in higher olfactory centers: the mushroom body (MB) and lateral horn (LH) of the protocerebrum. In the LH axon branching patterns of PNs of the same glomerular class were highly stereotyped across animals, while such stereotypy was less evident in the MB. Tanaka et al. (2004) have described several putative output neurons of the LH. Understanding how these neurons integrate olfactory information is a key problem in the neural basis of olfactory perception. In mice, the existence of some spatial organization in higher olfactory centers has been reported by following the targets of 2 of the 1000 ORN classes to the olfactory cortex (Zou et al., 2001). The integrative properties of olfactory cortical neurons have also been studied (Zou and Buck, 2006). However, the anatomical basis of this integration remains challenging because of the numerical complexity of the rodent olfactory system.

Neuroanatomy is the foundation of both developmental and functional studies of the brain. In order to understand the development of neuronal wiring, it is necessary to describe the degree of wiring precision across individuals. Similarly, high-resolution neuroanatomy makes predictions about information transfer and transformation, constraining models of neural processing. Two anatomical approaches have been particularly influential in constructing wiring diagrams. The first is exemplified by the classic work of Cajal (1911) using the Golgi method. A small fraction of the neurons within a piece of tissue are stained to reveal their dendritic and axonal projection patterns; the information from many specimens is compared and integrated to give a global picture of the circuit. While this approach was enormously successful in defining the basic logic of connectivity, it lacks comprehensiveness and precision: comprehensiveness because only a small fraction of the neuronal elements are used to construct the global picture; precision because integrating information across sample brains has allowed only qualitative comparisons. The second method is a complete reconstruction of all the connections in a small number of specimens through serial electron microscopy. While new EM technologies are under development (Denk and Horstmann, 2004), traditional serial section transmission EM approaches are so labor intensive that this has only been achieved once—the reconstruction of the nervous system of *C. elegans* hermaphrodites (White et al., 1986).

Here, we describe an approach that has merits of both methods. By combining genetic single-cell labeling with state-of-the-art image registration techniques, we have produced comprehensive maps of the LH and MB—the two higher olfactory centers of *Drosophila*. We can therefore visualize and directly compare projections of individual neuronal classes with their neighbors. These three dimensional maps directly demonstrate the spatial stereotypy of input to the LH and MB. We have derived probabilistic synaptic density maps and used them to identify and quantify the organizational principles of these two centers, finding, for example, that fruit odors and pheromones are represented in distinct compartments of the LH. Finally we have characterized postsynaptic neurons of the LH at the single-cell level and used our density maps to predict connectivity with input PNs. All the raw and derived data and the necessary software tools are available on the project website, providing a resource that will be integrated with future anatomical, physiological and behavioral data to understand the neural basis of olfactory perception in *Drosophila*.

## Results

### Image Registration

The results we are about to present depend critically on an accurate method for bringing images of different specimen brains into a common reference space. We used a fully automatic, nonrigid, intensity-based 3D image registration algorithm (Rohlfing and Maurer, 2003). In addition to linear transformations, where an image is globally scaled, translated, and rotated to match a template, this algorithm also utilizes nonrigid deformations (warping) that allow different parts of the brain to deform to different extents. We applied this algorithm to two-channel confocal images of adult *Drosophila* brains. The green channel typically contained a single fluorescently labeled neuron generated by MARCM (Lee and Luo, 1999), while the magenta channel contained monoclonal antibody nc82 staining, a presynaptic marker which reveals brain architecture (Figures S1 and S2). The nc82 channel for each specimen was registered with an nc82 stained reference brain (Figure S1) that includes the MB calyx and LH. As outlined in Figures 1B and 1C, the sample brain is progressively registered with the reference brain by rigid and nonrigid steps.

257 successfully registered brains contained clearly identifiable single labeled neurons used in subsequent analyses. These include 236 PNs from 35 glomerular classes, including 11 classes whose axon branching patterns have not previously been described even qualitatively (Table S1; Figure S2); the remaining 21 neurons comprise three groups of LH neurons. Each neuron was traced, then transformed into the reference brain coordinate system by applying the registration calculated with the nc82 channel of that same specimen. Critically, both image registration and subsequent assessment of the registration quality used only the nc82 data. Therefore information from the labeled neuron could not assist the registration or bias our estimate of registration accuracy.

Previous single axon tracing experiments revealed that PNs have highly stereotyped branching patterns within the LH according to their glomerular classes (Marin et al., 2002; Wong et al., 2002). With our new registration-based technique, we can now directly visualize and compare the projections of single axons of different PN classes within the same coordinate system. Figure 1D_1_ illustrates 35 singly labeled axons before registration, 5 neurons from each of 7 PN classes, each class labeled in a different color. After rigid (Figure 1D_2_; Movie S1 in the Supplemental Data available with this article online) and subsequent nonrigid (Figure 1D_3_; Movie S2) registration, the spatial stereotypy of PN projections is demonstrated by the segregation of differently colored axons into distinct groups. Movie S3 provides a 3D visualization of the axonal projections of different PN classes within the standard MB and LH while two-dimensional (2D) representations of single specimens from all 35 PN classes are shown in Figure S3.

### Registration Accuracy

We have calculated two separate estimates of registration accuracy. These estimates include two sources of variability: the error associated with sample processing and the algorithm, and the biological variation in the structure and relative position of neuronal processes within each different brain.

Figure 1E plots 40 different PNs from four glomerular classes. Each PN has a major branch point shortly after entering the LH. We model branch point position by a class-specific mean plus a standard deviation, which corresponds to the registration accuracy that we wish to estimate. We find values of 2.64, 1.80, and 2.81 μm, respectively, for the three axes (Figure 1F), indicating that the performance of our registration algorithm is at least this good, even assuming no biological variation. Indeed we made a new biological observation: we could statistically separate the branching points of the 4 PN classes (Supplemental Data), demonstrating the high degree of spatial stereotypy of PN axon branching patterns.

A second piece of evidence supporting our registration accuracy is a novel observation about axon positions of PNs of different developmental origins in the inner antennocerebral tract (iACT) that joins the antennal lobe to higher olfactory centers. After registration we found that axons segregate within the iACT (10–15 μm in diameter) according to their birth time and lineage: embryonic-born PNs (Marin et al., 2005) are clearly separated from larval-born PNs (Jefferis et al., 2001), which are further segregated according to their parental neuroblast (Figure 1G). A statistical analysis confirmed a significant difference in the mean position of these three axon bundles even though the centers of the two closest axon bundles were only 3.4 μm apart in the reference brain. Indeed the axon position within the iACT of a PN of a given glomerular class can be predicted with a standard deviation of ±2.5 μm in each axis (Supplemental Data), an accuracy level similar to that calculated for branch positions in the LH.

In summary, these examples give an upper bound on the registration error of a few microns.

### Location of PN Presynaptic Terminals

**Figure 2:**
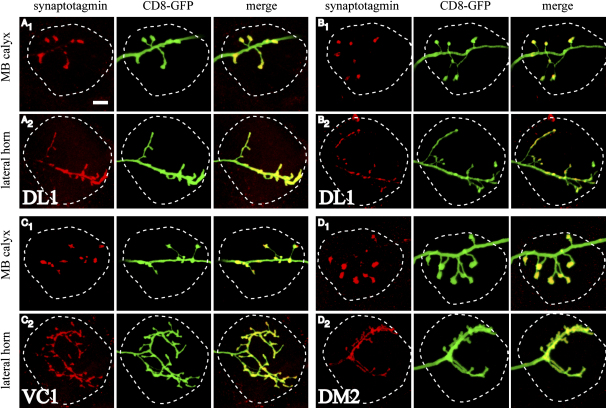
Distribution of Presynaptic Terminals on Single PNs Localization of presynaptic reporter UAS:synaptotagmin-HA (red) in the MB calyx (A_1_–D_1_) and LH (A_2_–D_2_) of four single-cell PN MARCM clones. Glomerular classes and labels as indicated. Merged images show localization of presynaptic termini on the axonal projections (yellow). Outlines for the MB calyx and LH are based on nc82 staining (data not shown). The scale bar represents 10 μm.

To determine the sites of olfactory information transfer from PNs to their postsynaptic partners, we generated single-cell MARCM clones expressing membrane tagged GFP to label axons (Figure 2, green), and synaptotagmin-HA to label presynaptic terminals (Figure 2, red). In all cases, synaptotagmin-HA localized only to the terminals or occasional large varicosities of axonal arbours in the MB calyx (Figures 2A_1_–2D_1_). In contrast, the same labeled PNs had synaptotagmin-HA localized through most of their LH axonal arbours (Figure 2A_2_–2D_2_). These findings applied to all PN classes examined (Figure S4). Examination of multiple single-cell samples of the same class did not reveal a stereotyped location of synaptotagmin-HA puncta within the LH. Thus, to a first approximation, the entire LH axonal arborization can be considered a possible site of synaptic transmission.

### Quantitative Maps of PN Synaptic Density in Higher Olfactory Centers

The synaptic distribution data allowed us to transform PN axon maps into density maps, which describe the probability of finding presynaptic terminals of a particular class of neuron at each point in space in the LH and MB calyx.

**Figure 3:**
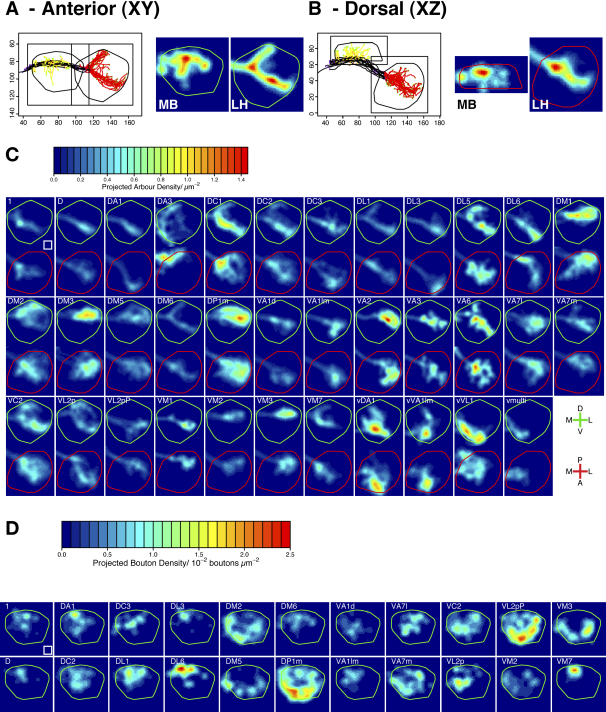
Presynaptic Density Maps of PNs in the LH and MB (A and B) Example of conversion of tracings to 3D density map using 11 DM6 PNs. (A) shows a standard anterior view and (B) shows a dorsal view, with posterior uppermost. The outlines of the MB (middle subpanel) and LH (right subpanel) are shown in green for anterior (A) and red for dorsal (B) views in this and all subsequent figures. Axis units are μm. (C and D) 2D projection of LH arbour density (C) and MB bouton density (D) data, integrating along the Z (anterior-posterior) axis or Y (dorsal-ventral) axis. Axes for the anterior view (green outline) and dorsal view (red outline) are shown in the bottom right of this panel. Abbreviations are as follows: D, dorsal; V, ventral; L, lateral; M, medial; A, anterior; P, posterior. These axis markers recur in subsequent figures. White square represents 100 μm^2^.

In the LH, we assume that synapses occur with a fixed probability along each linear micron of axon arbour. Our analysis follows the approach of Stepanyants and Chklovskii (2005) by calculating an arbour density, defined as the length of axonal arbour that crosses each unit volume in space. Figures 3A and 3B illustrates the conversion of PN tracings into a density map. Figure 3C presents such results for 35 PN classes in the LH. The 3D density has been projected down into two dimensions in the anterior (XY) and dorsal (XZ) planes, with the LH border outlined in green or red, respectively. All density data are plotted on the same absolute scale, so that the contributions of different PN classes to a given LH region can be directly compared.

In the MB calyx we assumed that presynaptic terminals are located at the tips of the MB collaterals (Figure S5). It is clear from the resultant density maps that individual PN classes can project to quite discrete locations in the MB calyx (Figure 3D), a stereotypy not detected in earlier studies (Marin et al., 2002). The difference is likely accounted for by the high resolution of our new registration approach. In addition, the stereotypy appears statistical: individual PNs of the same class have somewhat similar MB branching patterns, but when examined together, there are regions of the calyx in which they are highly likely to form terminals. The density maps therefore highlight stereotypic axon terminal locations that can be hard to visualize in the initial branching maps. For example, D, DL3, and VM7 PNs have very focal projection patterns. DM5 and VM3 PNs project to the edge of the calyx, displaying a 4-fold symmetry that appears to correspond with the dendritic organization of their postsynaptic partners: MB neurons belong to one of four symmetric clones each derived from a single neuroblast; the dendrites of each clone are restricted to a quarter of the MB calyx (Ito et al., 1997; Zhu et al., 2003; Strausfeld et al., 2003). Some PN classes do not seem to have terminals in all four subdivisions of the calyx, so we predict that the four clones of MB neurons may not receive equivalent odor input.

### Spatial Organization of Higher Olfactory Centers

We next used the density maps to investigate the overall spatial organization of PN input to the LH and MB. Because of the high dimensionality of our data, we employed two dimension-reduction schemes to visualize the organizational logic underlying the projections of different PNs. Both highlighted similar organizational features. We present results from independent components analysis in Figure S6 and focus below on results obtained from a cluster analysis (see Experimental Procedures).

**Figure 4:**
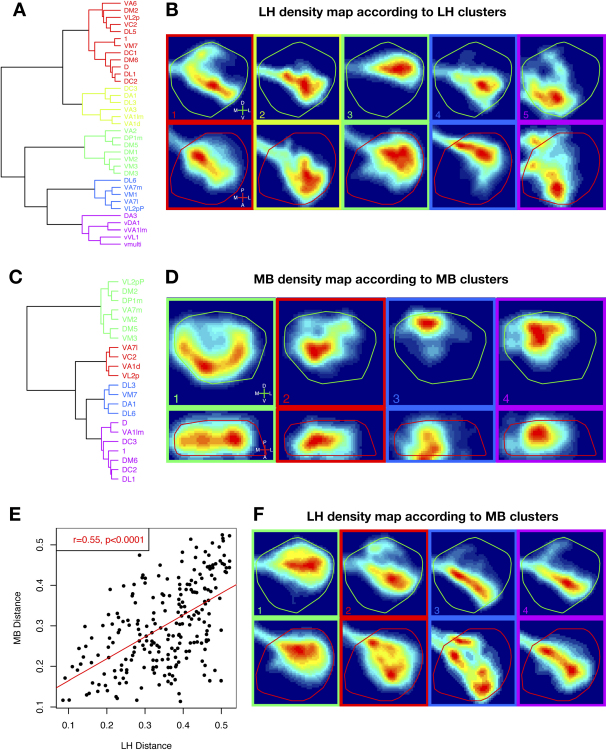
Organizational Principles of the LH and MB (A and C) Cluster analysis of PN axon projections in the LH (A) and MB (C). (B and D) Average density maps for PN clusters. The five clusters identified in (A) based on LH projection patterns are shown in (B) as five separate average density maps (both anterior and dorsal views) with a colored box and number identifying each corresponding cluster. The procedure is repeated in (D) for the MB clusters. (E) Correspondence of the spatial organization in LH and MB. Each point in the XY plot represents a distance between the density maps of two neuronal classes in the MB calyx plotted against the corresponding distance between the LH density maps for the same pair of classes. 22 PN classes generate 231 pairwise distances. The correlation coefficient, r, was calculated from these values and the p value then corrected for the data's multiple correlation structure by a 10,000 permutation Mantel test. (F) Evidence that PN innervation of the MB calyx and LH may be coordinately organized. The 4 PN clusters identified on the basis of projection patterns in the MB calyx (see [C] and [D]) are replotted to show the corresponding average densities in the LH.

The LH dendrogram divides PNs into 5 major clusters (Figure 4A); the average LH projection patterns for neurons in these clusters are plotted in Figure 4B. The first four clusters consist of conventional PNs (iPNs) whose axons take the iACT to the MB and LH. These clusters divide up most of the LH with little spatial overlap between clusters. The fifth cluster is distinctive, corresponding mostly to PNs with ventral cell bodies that take a path directly to the LH though the middle antennocerebral tract (mACT), bypassing the MB. Axons of these vPNs terminate in the ventromedial corner of the LH, which is avoided by iPN projections. This cluster also includes an iPN class, DA3, which is unusual in having axon terminals that never enter the LH and en passant collaterals that fail to enter the MB calyx (Figures S2 and S3). The LH can therefore be divided into a number of spatial sub-regions on the basis of PN projection patterns. We describe our analysis of the biological significance of this organization later.

For the MB, we identified four clear clusters from 22 PN classes (Figures 4C and 4D). Each domain is approximately radially symmetric and can be characterized by the length of the contributing axon collaterals—collateral length increases in the order: cluster 3, 4, 2, and 1. Cluster 3 PNs appear to have a focal density map with very short collaterals that barely leave the iACT axon tract. In contrast, cluster 1 collaterals terminate in a ring near the edge of the calyx, and the constituent neurons in some cases exhibit clear 4-fold symmetry in their projections.

### Relationship between Gross Spatial Organization of MB and LH

A comparison of the dendrograms in Figures 4A and 4C suggests a number of cases in which PNs that have similar projections in the LH also have similar projections in the MB. This correspondence was supported by statistical comparison of the two distance matrices for PN classes in the LH and MB calyx (Figure 4E).

To directly visualize this spatial correspondence, we took the four clusters identified on the basis of PN projection patterns in the MB (Figure 4C) and computed the average density of the corresponding PN axon terminals in the LH (Figure 4F). This analysis reveals that MB cluster 1 PNs, whose long collaterals occupy the periphery of the MB calyx, have projections in the dorsal posterior region of the LH, resembling LH cluster 3 PNs (Figures 4A and 4B). Indeed there is significant overlap in the membership of LH cluster 3 and MB cluster 1. While there is not a simple one-to-one correspondence for all clusters, there is a general rule: termination zones schematized as concentric circles from inner to outer MB calyx correspond to a progression from the anterior-ventral to posterior-dorsal LH.

### Biological Correlates of Higher Olfactory Center Organization

Recent progress in *Drosophila* olfaction has established a nearly complete map of the projections of ORNs expressing specific odorant receptors to the identified glomeruli of the antennal lobe (Couto et al., 2005; Fishilevich and Vosshall, 2005). By combining this information with our PN map, we can correlate the organization of higher order olfactory centers directly with different ORN input channels.

**Figure 5:**
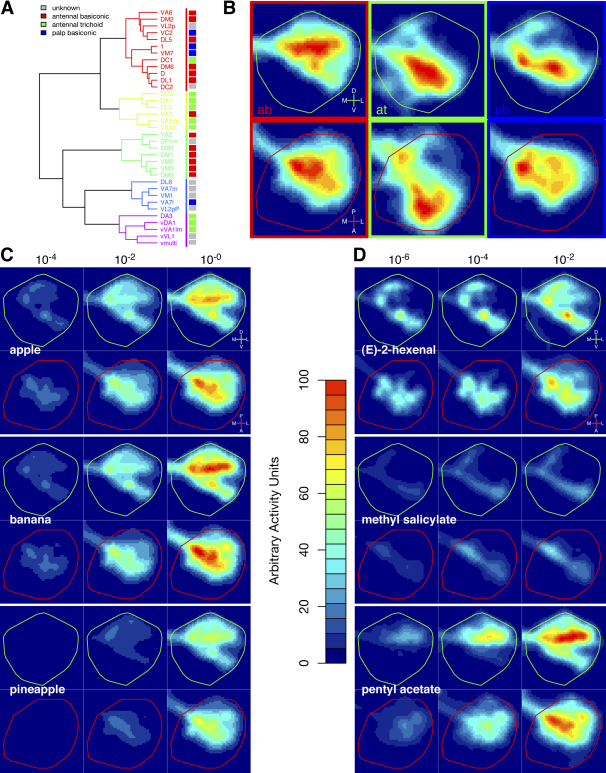
LH Representation of Sensillar Groups and Simulated Odor Input (A) The dendrogram in Figure 4A is replotted with the sensillar group of the ORNs that project to each glomerulus indicated to the right of the figure (colored boxes). (B) PNs originating from glomeruli innervated by ORNs from different sensillar classes have distinctive spatial projections in the LH. Anterior (LH outlined in green) and dorsal (red outline) views are shown for each sensillar category. (C and D) Predicted LH odor response profiles. The anterior (green) and dorsal (red) LH activity maps are linear superpositions of the axonal density map for a particular PN class multiplied by the firing rate of the corresponding odorant receptor to three fruit extracts (C) and three odorants (D) at specified dilutions. Activity units are arbitrary (heatbar), but the scales are identical for all odorants and all fruit extracts.

*Drosophila* ORNs are found in two sensory organs, the antenna (∼90%) and the maxillary palp. In the antenna, ORNs of each class reside in one of three broad types of sensillum: basiconic, trichoid or coeloconic. Each sensillum type has a distinct morphology, spatial distribution and function (Stocker, 2001). Incorporating sensillar information into the dendrogram (Figure 5A), we find strong correlations between sensillum class and different LH clusters. For instance, the vast majority of antennal basiconic ORNs constitute two major LH clusters (1 and 3), while two other LH clusters (2 and 5) represent trichoids (Figure 5A). Maxillary palp basiconics mostly fall into cluster 1. Plotting all LH inputs from PNs that receive information from antennal basiconic, trichoid or maxillary palp basiconic sensilla yields three distinct density maps, with the difference between trichoids and other classes especially clear (Figure 5B). Although the palp and antennal basiconic maps overlap, palp basiconics project to a significantly restricted area, sparing the dorsolateral corner of the LH.

The odor response profiles of 24 antennal and all 6 maxillary palp ORN classes have been reported (Hallem and Carlson, 2006). In general, fruit odors activate antennal and maxillary palp basiconic sensilla but not trichoid ORNs, whereas trichoid sensilla house pheromone sensitive ORNs in moths (Kaissling et al., 1978) and flies (see later). Thus LH organization may correlate with different biological inputs. We carried out a parallel analysis for the MB calyx (Figure S7) but with the available data found no organizational correlations.

### Simulating Odor Input to the LH

The PN synaptic density map defines the spatial transformation of odor information from the antennal lobe to the LH. We have combined this with physiological data from Hallem and Carlson (2006) to simulate putative “odor activation maps”. We have adopted the simplest model as a starting point: the response to a given odor at any position in the LH is proportional to the weighted sum of the PN densities at that position multiplied by the firing rate of each corresponding ORN class to that odor.

The results are plotted for three fruit extracts (Figure 5C) and three pure odorants (Figure 5D) at three different concentrations. Apple, banana and pineapple all activate a large fraction of the LH. However this activation is restricted to the dorsal and posterior LH, corresponding strongly to the average basiconic projection map (Figure 5B), sparing ventral and anterior regions that are targets of trichoid input. Comparing the three fruits, there are no large differences in the activation maps at high concentration. At lower concentrations the activation maps appear to be grossly similar but less intense. Based on the LH map alone, we speculate that these fruit extracts have broadly similar percepts across both different fruit types and concentration.

The three pure odorants show greater specificity in their activation maps. Pentyl acetate, an odorant that smells strongly of banana to humans, recapitulates the banana pattern at high concentration (Figures 5C and 5D). At the intermediate and lower concentrations the patterns are somewhat distinct, showing a more restricted focus in a dorsal and posterior region. The other odorants have much more restricted activation maps corresponding to a more selective action on particular ORN classes across a wide concentration range—this is particularly true of methyl salicylate (highly selective for Or10a ORNs projecting to DL1). (E)-2-hexenal also shows a restricted map, which broadens out to approach the “fruity” pattern at high concentration (Figure 5D). Interestingly, (E)-2-hexenal is commonly described as having characteristics of apple and unripe banana; at low concentrations (10^−6^) the (E)-2-hexenal activation pattern is rather similar to low concentrations of banana and especially apple extracts but not pineapple extract (Figures 5C and 5D). Thus pure odorants can vary widely in the extent to which they activate different portions of the LH and should be distinguishable on the basis of these activity patterns.

This particular simulation assumes that PNs only receive odor information from ORNs that project to the same glomerulus in the antennal lobe. While there is experimental support for this labeled-line hypothesis (Ng et al., 2002; Wang et al., 2003), significant broadening of PN tuning profiles compared with their corresponding ORNs has also been reported (Wilson et al., 2004). Regardless of the outcome of this controversy, our simulation gives initial predictions for odor activation maps based on our anatomical findings. When a more substantial library of PN physiological data becomes available, the same technique can be used to generate a more exact functional prediction of PN input to the LH and MB.

### Spatial Segregation of Pheromone Representation in the LH

Recent studies of *fruitless* (*fru*), a master regulator of male sexual behavior, have identified olfactory channels likely to process sex pheromones during male courtship (Demir and Dickson, 2005; Manoli et al., 2005; Stockinger et al., 2005). Fru is expressed in a subset of trichoid ORNs that project to three glomeruli whose volume is sexually dimorphic. Blocking synaptic transmission in these ORNs profoundly reduced male courtship (Stockinger et al., 2005). These ORNs, which project to glomeruli DA1, VA1lm and VL2a, therefore represent candidate channels for sex pheromones.

**Figure 6:**
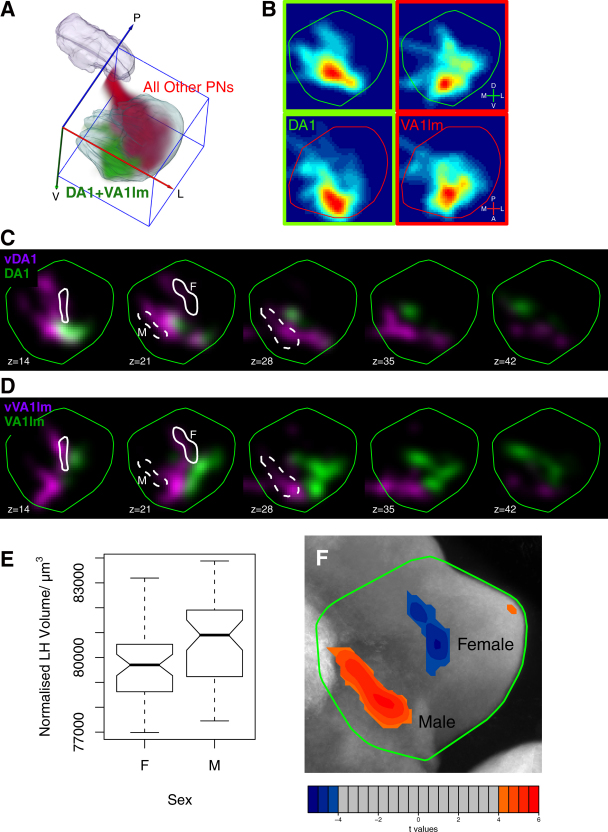
Pheromone Representation and Sexual Dimorphism (A) 3D rendering of axonal projections in the LH for DA1 and VA1lm PNs that contact glomeruli of Fru+ ORNs (green) compared with the sum of all other PN classes (red). Note the complementary positions. (B) PNs contacting Fru+ ORNs occupy an anterior ventromedial position in the LH. Shown are synaptic density plots for the combinations of vDA1 and DA1 PNs, and vVA1lm and VA1lm. (C and D) Comparison of exact projections of putative excitatory (green) and inhibitory (magenta) DA1 (C) and VA1lm (D) PNs shows regions of overlap (white) and significant nonoverlap. Sexually dimorphic regions identified in (F) are outlined; M and F indicate, respectively, the male (dashed white line) and female (solid line) enlarged regions. Both regions overlap with vDA1 and vVA1lm PNs. Each image is a frontal section (anterior view) at the indicated Z depth (μm). Standard LH outline in green. (E) Sexual dimorphism of LH volume normalized with respect to our reference brain. Central line median; notches, 95% confidence interval for difference between medians; box, 25% and 75% centiles; whiskers, ±1.5 × the interquartile range. The notches do not overlap, indicating a significant difference in median male and female LH volume (p < 0.05). (F) Mapping sex-specific LH volume differences. An anterior view of the volume difference t-statistic map after maximum intensity projection and thresholding at t = ±4.07 (p = 0.05 level). Colored scale bar shows range of t values: negative (blue), female-enlarged region; positive (red), male-enlarged region. Statistically significant male- and female-enlarged regions are also outlined in panels (C) and (D). See Figure S11 for additional volumetric analyses.

We find that axons of DA1 and VA1lm PNs project to a distinctive anterior-ventromedial region. (VL2a is not among the 35 PN classes analyzed.) Interestingly, fruit odors analyzed in the previous section do not activate this LH region. In fact, projections of these candidate pheromone PNs appear complementary to almost all other PN projections (Figure 6A). Specifically, VA1lm PNs only show additional overlap with VA1d and D PNs; for DA1, only DL3 shows significant overlap. Thus the PNs that overlap with LH regions receiving Fru+ ORN input are PNs that innervate adjacent glomeruli and are usually also targets of trichoid ORNs (compare Figure 6B with Figure 5B). This observation strongly suggests that a spatially segregated representation of pheromones persists from the antennal lobe to the LH.

Interestingly, VA1lm and DA1 are the only two glomeruli dually represented by conventional iPNs and by vPNs that project directly to the LH via the mACT. While most PNs are cholinergic and excitatory (e.g., Yasuyama et al., 2003), a recent study reported that GH146+ ventral PNs are GABA-positive (Wilson and Laurent, 2005) and likely to be inhibitory. We have confirmed with single-cell resolution that GH146+ vDA1 and vVA1lm PNs are GABA-positive (Figure S8). Close examination indicates that DA1, vDA1, and vVA1lm show significant but partial overlap in all pairwise combinations while VA1lm, although adjacent, does not overlap with the other 3 PNs (Figures 6C and 6D). This arrangement may allow target neurons to respond to all combinations of activation of these two glomeruli or to allow one signal to inhibit the response to another.

### Volumetric Sexual Dimorphism in the LH Correlates with Pheromone Processing Areas

Having shown that PNs originating from sexually dimorphic glomeruli target discrete LH regions, we next asked whether there are regional volume differences in the LH between the sexes and which PN axons target these regions. Nonrigid registration generates a deformation field that maps arbitrary points between each sample and the reference brain; this can be used to compare volumes of brain regions between sexes.

After normalising each brain to remove absolute differences in size, we found significant difference in the relative size of the LH (Figure 6E; 1% larger in males than females; p = 0.01, Wilcoxon test) and MB calyx (Figure S9; 15% larger in males than females; p = 0.001). We then carried out a voxel-wise analysis using a t-statistic parametric map to locate the volume differences in the LH (Figure 6F) and MB (Figure S9). We identified two small male and female enlarged regions corresponding to 3.0% and 1.6% of the total LH volume; both were 12% enlarged compared with the opposite sex. Technical limitations mean that the exact location of these voxels should not be over-interpreted (Supplemental Data) but this is strong evidence for dimorphism in these regions. The male enlarged region overlaps with vPNs; the 4 vPN classes that we mapped are the only PNs that overlap with >50% of this region. The female-enlarged region shows >50% overlap with a larger fraction of PN classes (12/35) including vDA1 and vVA1lm. Interestingly, the only two classes whose axon arbours overlap both male and female enlarged regions are the vPNs whose dendrites contact Fru+ ORNs in the DA1 and VA1lm glomeruli.

### Projections of LH Neurons at Single-Cell Resolution

To understand the anatomical basis of integration of PN input by candidate output neurons of the LH (LHNs), we examined their dendritic arborizations. We generated single-cell clones from three Gal4 lines labeling specific LHNs, and then registered the sample brains to our reference (Figure S10).

**Figure 7:**
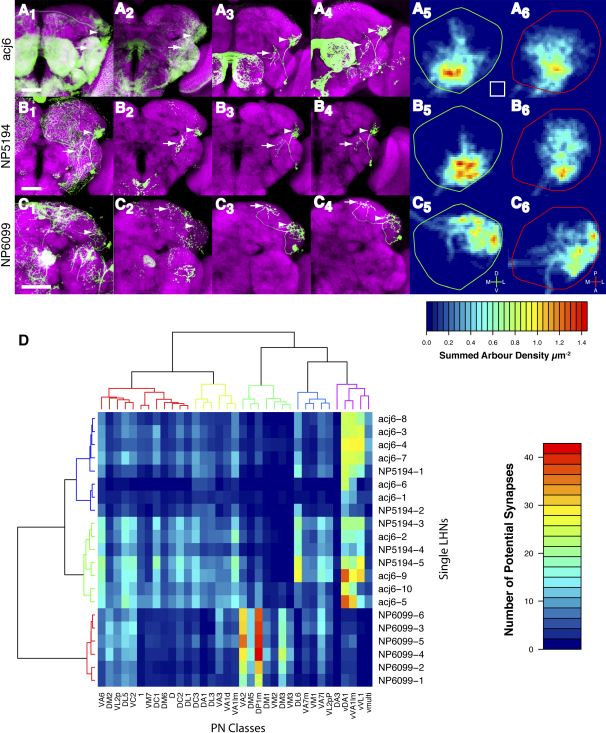
Characterization of LH Neurons and Their Potential Connectivity with PNs (A_1_–C_1_) Expression patterns of acj6-GAL4, NP5194-GAL4, and NP6099-GAL4-driven mCD8-GFP are shown in green. Magenta, nc82 staining. Arrowheads in all panels denote putative dendrites and arrows denote putative axons. The scale bar represents 50 μm. (A_2_–C_2_) UAS:Synaptotagmin-GFP localization is shown for three Gal4 lines as indicated. Only confocal slices with LHN innervation were used for Z projections. Note higher ratio of synaptotagmin to mCD8-GFP staining for LHN processes outside LH (arrows). (A_3_–C_4_) Two example single-cell MARCM clones of acj6-GAL4 (A_3_ and A_4_), NP5194-GAL4 (B_3_ and B_4_), and NP6099-GAL4 (C_3_ and C_4_). (A_5_–C_6_) Estimated density maps for each LHN class are shown for anterior (A_5_–C_5_) and dorsal (A_6_–C_6_) axis. Heat bar indicates calibrated arbour density in μm^−2^. White square represents 100 μm^−2^. (D) Potential connectivity of PNs and LHNs. PN classes (columns) are each the average of 1–14 neurons ordered according to the clustering defined in Figure 4A. Individual LHNs (rows) are clustered using a Euclidean distance calculated from the matrix of potential synapse numbers.

The first Gal4 line we characterized is acj6-Gal4 (Suster et al., 2003). The Acj6 transcription factor regulates odorant receptor expression (Clyne et al., 1999) and axon targeting in different ORN subsets (Komiyama et al., 2004) along with dendritic targeting and axon arborization of some PNs (Komiyama et al., 2003). Careful inspection of the acj6-Gal4 expression pattern (which closely mimics acj6 protein expression, data not shown) identified a ventral region of the LH innervated by neurons other than Acj6+ PNs. MARCM analysis of acj6-Gal4 identified a cluster of 7-8 LHNs whose cell bodies are in the anterior lateral region of the brain (Figures 7A_3_ and 7A_4_). These neurons send one projection to the ventromedial LH, and another to the ventrolateral protocerebrum (VLPR). To find the direction of information flow, we examined the localization of the presynaptic marker synaptotagmin-GFP (Figure 7A_2_). Although both processes were labeled by synaptotagmin-GFP, much stronger labeling was observed in the VLPR (arrows in Figure 7A; data not shown) suggesting that information flow is likely to be from the LH to VLPR.

Six Gal4 lines have been reported that may label LHNs (Tanaka et al., 2004). MARCM analyses identified two lines that consistently labeled single neurons with a process in the LH and a process in another brain region (Figures 7B and 7C). NP5194-Gal4 is expressed in 5 LHNs that have similar projections to acj6 LHNs (Figure 7B), but are a separate population (Supplemental Data). Synaptotagmin-GFP again localizes to both processes, but is enriched in the VLPR, suggesting that these are axonal arborizations (arrow in Figure 7B_2_). The LH density maps of NP5194 and acj6 are indeed similar although NP5194 LHNs seem to have slightly more lateral arborization (compare Figures 7A_5-6_ and 7B_5-6_).

Finally, NP6099-Gal4 is expressed in 2-3 LHNs with cell bodies anterior-ventral to the LH. They send projections to the dorsolateral LH and superior lateral protocerebrum (SLPR; Figures 7C_1_ and 7C_3-4_). Synaptotagmin-GFP is relatively enriched in the SLPR, suggesting that this branch is the axonal process (arrow in Figure 7C_2_). Density maps indicate that NP6099 neurons target a dorsolateral region of the LH, distinct from acj6 and NP5194 LHNs (Figure 7C_5-6_).

### Potential Connectivity in the LH

Having mapped the position of PN axons and putative LHN dendrites, we can ask which classes of these candidate partners are likely to form synapses with one another. We defined potential synapses as axo-dendritic separations of <1 μm, a criterion that is necessary but not sufficient for a real synaptic connection (see Experimental Procedures). The average number of potential synapses between each PN class and single LHNs varies widely (range 0–50; Figure 7D).

All six NP6099 LHNs are in a cluster distinct from all NP5194 and acj6 LHNs and show a very similar profile of potential connectivity. In contrast, acj6 and NP5194 LHNs do not form separate clusters and heterogeneity (especially for acj6) is evident in the individual tracings in Figure S10. Intriguingly, the overall picture for acj6 and NP5194 LHNs is that, although they may integrate across a large number of PN classes, they receive their strongest predicted input from the vPNs, vVA1lm, vDA1 and vVL1. Some of these LHNs may also receive appreciable levels of input from the Fru+ iPN VA1lm (e.g., acj6-2 and NP5194-5) and therefore have the opportunity to integrate both excitatory and inhibitory input from this glomerulus. It will be very interesting to see how this potential connectivity is translated into functional connectivity.

## Discussion

We have combined single neuron labeling and image registration methods to provide a comprehensive description of the organization of higher olfactory centers in the *Drosophila* brain. We discuss our methodology (which may have general applications), the spatial organization of the olfactory system, and the biological implications of these results.

### Constructing Wiring Diagrams by Registering Single Neurons to a Common Brain

Deciphering neuronal wiring diagrams is essential to understanding the organization, development and function of the nervous system. A classic example is the almost complete EM reconstruction of neuronal connectivity of the *C. elegans* hermaphrodite (White et al., 1986), which has had a great impact on studies of neuronal development and more recent functional studies of the circuitry underlying behavioral processes (Schafer, 2005). In vertebrates, much of our knowledge of neuronal wiring diagrams derives from compilations of Golgi stained samples that reveal dendritic and axonal projection patterns of single neurons of different types (Cajal, 1911), and bulk labeling that traces coarse connections between different brain regions (Cowan, 1998). Only in highly organized brain regions (e.g., retina and cerebellum) do we have a more comprehensive understanding of microcircuits. The spatial architecture of other brain regions such as the neocortex is much less defined and it is rarely possible to make definitive statements about connectivity from independent anatomical samples. In the olfactory system, ORN axons and PN/mitral cell dendrites target discrete glomeruli in the first relay station; connectivity can be inferred relatively easily from independent samples. However, this regular organization gives way to an apparently unstructured organization in higher olfactory centers. Understanding the organization of this kind of neuropil represents both a major technical challenge and a major goal in understanding the integrative properties of the nervous system; higher brain functions largely depend on brain centers that have an apparently looser structural organization.

While serial EM reconstruction could be used to obtain a complete wiring diagram of small pieces of nervous tissue, it becomes very difficult for more complex tissues with long-distance axon projections. The combination of single neuron labeling and image registration we describe here represents a useful intermediate: it allows the substructure of an information-processing center to be defined according to input and output channels and predicts their synaptic relationships. We show that the registration accuracy of identifiable PNs is of the order of 2.5 microns even assuming no biological variation. This has allowed us to demonstrate the high level of spatial stereotypy of PN axon branching, terminal arborization and axon position within axonal fascicles.

Although rigid image registration has been used to align large structures within the fly brain (Rein et al., 2002), our nonrigid, intensity-based automated registration method is more suitable for high-resolution study of individual brain structures. This study focused on higher olfactory centers, but our approach is applicable to any brain region that contains sufficient structural information for registration. Detailed connectivity maps could be constructed for the neurons of other complex neuropils, such as the optic lobe and central complex. Furthermore, information from studies carried out in different laboratories can be integrated by co-registering future data to publicly available reference brain regions, creating a growing repository of neuronal connectivity information, ultimately generating a detailed model of the major neuronal components of the fly brain. We believe that these models will be a crucial intermediate in the construction of synapse by synapse wiring diagrams of the fly brain.

Since the registration algorithm is not species specific, our methodology could be applied to nervous tissue in other organisms where one can collect a large number of single neuron samples counterstained with a neuropil marker. Single neuron labeling can be achieved by the Golgi method, genetic methods (Young and Feng, 2004; Zong et al., 2005) or by filling neurons during single-cell recording. Additionally, application to quantitative analysis of wiring defects in mutant neurons will contribute to our understanding of the molecular mechanisms of neural circuit assembly.

### Organization of Higher Olfactory Centers in *Drosophila*

Previous studies have revealed aspects of the spatial organization of higher olfactory centers—the MB calyx and the LH. Of particular relevance to the principles of olfactory information processing, Marin et al. (2002) and Wong et al. (2002) found that single PNs of different classes have highly stereotyped LH projections. Using five Gal4 enhancer trap lines each labeling 1-3 PN classes, Tanaka et al. (2004) found that PNs from 9 glomeruli project to 3 corresponding zones in the MB calyx and LH; MB output neurons integrate information from each of these zones whereas 6 groups of putative LH output neurons maintain the segregation of these 3 zones.

This study contains several advances. First, we described the projection patterns of 11 new PN classes at single-cell resolution (Figures S2 and S3), qualitatively extending previous results (Marin et al., 2002; Wong et al., 2002). Second, we digitized all single neuron tracings, transformed them onto a common reference brain. Third, we determined, at the single neuron level, the distribution of PN presynaptic terminals in the MB and LH. Fourth and most importantly, combining the above information allowed us to generate quantitative synaptic density maps for 35 PN classes, representing 32 of ∼50 unique olfactory channels defined by the projection of ORN classes to antennal lobe glomeruli. This allowed us to decompose MB and LH input into individual channels and then reassemble them for most of the olfactory system, providing a global view of these higher order centers. Lastly, we also described projection patterns of three groups of LHNs at single-cell resolution, and made predictions about their physiological properties based on their potential connectivity with specific PN classes.

We quantitatively confirmed the concentric zonal organization of PN input into the MB calyx proposed by Tanaka et al. (2004). However, LH organization is more complex and cannot simply be described as zonal as proposed based on data for a limited set of PN classes (Tanaka et al., 2004), with the exception of the segregation of pheromone projections from the rest of the channels. This is evident from the single neuron projections of many classes (Figures S2 and S3) that send stereotyped and divergent branches to multiple areas of the LH, as well as the synaptic density maps (Figure 3C). Together with the extensive branching of individual LHNs (Figure 7), characterizing the LH as providing relatively little integration across glomeruli (Tanaka et al., 2004) is inaccurate.

Comparing PN branching patterns in the LH and MB suggests that the LH is likely to support more stereotyped integration. This proposal is consistent with the view that the LH mediates innate olfactory behaviors while the MB participates in odor-mediated learning (Heimbeck et al., 2001; Heisenberg, 2003). However, we have now demonstrated a clear stereotypy of PN terminals in the MB calyx. This is likely to explain the observations of Wang et al. (2004) that certain odors can evoke spatially stereotyped activity in MB neurons. Thus the MB calyx and LH receive different levels of stereotyped input that can be integrated by third order coincidence detectors (Perez-Orive et al., 2002; Zou and Buck, 2006) that combine information from different input channels.

### Biological Implications and Future Predictions

The most striking biological insight we have obtained from this study is the segregation in the LH between putative pheromone representing PNs and almost all other PNs in the apparently homogeneous LH neuropil (Figure 6A). Interestingly, the highest degree of LH volumetric sexual dimorphism that we quantified coincides with the presynaptic terminals of the GABAergic vVA1lm and vDA1 PNs. It is important to note that in addition to the GH146+ PNs that we characterized here, there may be other PNs that relay pheromone information from VA1lm and DA1 glomeruli to higher brain centers (Stockinger et al., 2005; Manoli et al., 2005) and contribute to the sexual dimorphism that we found in the LH.

The convergence of excitatory and inhibitory projections from these putative pheromone representing glomeruli at overlapping or adjacent locations may allow postsynaptic neurons to respond to the presence of a signal that activates these two glomeruli in a particular ratio or to allow signals from these two glomeruli to have opposing effects on LH neurons that initiate particular behaviors. Behaviorally, male flies appear to integrate information both from attractive and inhibitory pheromones produced by other males (e.g., Ferveur and Sureau, 1996; Stockinger et al., 2005). Furthermore, new data show that Fru+ Or67d ORNs innervating the DA1 glomerulus detect a male sex pheromone that has a negative effect on other males and a positive effect on females (Kurtovic et al., 2007). We speculate that balanced excitation and inhibition in these pathways may regulate LHNs that contribute to the appropriate behavioral alternative. Sex-specific integration in the lateral horn may underlie sex-specific behaviors.

The spatial segregation of pheromone representation contrasts with the representation of glomeruli that receive input from ORNs of the basiconic sensilla, which are generally activated by fruit odorants. Many of these PN classes have extensive overlap in their LH synaptic density maps (Figure 4B). This property, coupled with the fact that many fruit odorants activate multiple classes of basiconic ORNs (de Bruyne et al., 2001), makes the representations of different fruit odorants and natural fruit odors quite overlapping even if we assume the labeled line hypothesis (Figure 5). Our data thus support the following principles: olfactory information concerning food has extensive structural intermixing at the LH compared to the glomerular organization of the antennal lobe, but rather discrete channels are retained for pheromones all the way from the sensory periphery to the LH. We propose that the LH is globally organized according to biological values rather than chemical nature of the odorant information.

This finding is reminiscent of the male silkworm moth, *Bombyx mori*, where PNs from the macroglomeruli representing sex pheromones send axon projections to a discrete area in the lateral protocerebrum defined by a high level of anti-cGMP staining (Seki et al., 2005). Spatial segregation of the pheromone representation in higher olfactory centers may therefore be a conserved feature in insects. This segregation is exaggerated into two entirely separate pathways in mammals, where the nasal epithelium and main olfactory bulb process general odorants and some pheromones, while the vomeronasal organ and accessory olfactory bulb are more specific to pheromone sensation (Dulac and Wagner, 2006). Furthermore, mitral cells originating from the main and accessory olfactory bulbs project to distinct areas of the cortex (Scalia and Winans, 1975).

Having generated a comprehensive and quantitative map of PN input to the LH, a future challenge is to identify and characterize third order LHNs: where are their dendritic fields in the LH, with which PNs do they form synapses, where do they send their axonal outputs, and what are their physiological properties and functions in olfactory behavior? Tanaka et al. (2004) have started this effort by identifying Gal4 lines labeling neurons with projections in the vicinity of the LH. We have now characterized three groups of LHN at single-cell resolution and predicted their potential connectivity with different PN classes. However this is clearly only a beginning. The widespread distribution of LHN cell bodies and their potential output to different parts of the brain along with the difficulty of identifying large groups of LHNs labeled by new Gal4 enhancer traps (our unpublished observations) suggest that LHNs are heterogeneous genetically, anatomically and, in all likelihood, functionally. One tractable avenue will be to find LHNs that send dendrites to DA1/VA1lm PN target areas and may therefore respond to pheromones and instruct mating behavior. Two LHN groups that we characterized project to this LH region, and single-cell and potential synapse analyses indicate that some of these LHNs may form strong connections with pheromone responsive PN channels. Further characterization of these and other LHNs will bring us closer to understanding the neural circuit basis of olfactory perception and behavior.

## Experimental Procedures

Detailed Experimental Procedures are in the Supplemental Data. Software and data are at http://flybrain.stanford.edu.

### Reference Brain

This was an average of 16 co-registered brains. The initial seed brain was female; 13 additional female and 2 male brains were registered to this seed and then all 16 were averaged. The reference includes the dorsal posterior quarter of the left brain hemisphere, a volume of approximately 168 × 168 × 87 μm.

### Image Registration

Brain images were first roughly aligned to the reference using a linear registration with 9 degrees of freedom (translation, rotation and scaling in 3 dimensions), followed by a nonrigid registration. A parameter controlling the spatial smoothness of the warping was selected at a pilot stage to allow sufficient deformation for accurate registration while preventing unrealistic deformations.

### Tracings and Density Maps

Single neurons were manually traced from confocal stacks, then transformed to the reference coordinate system. In the LH, tracings for each neuronal class were convolved with a 3D Gaussian kernel with 2.5 μm standard deviation to generate a smoothed density estimate of the number of terminals in each region of space. For the MB, positions of individual boutons for each PN class were convolved with a kernel with σ = 3.5 μm.

### Spatial Organization via Cluster Analysis

We first calculated a distance matrix between different classes of neuron in the LH or MB based on the similarity (Pearson correlation coefficient) of their density maps, then used this matrix as the input for clustering by Ward's algorithm. The MB and LH distance matrices were compared using a Mantel test to look for organizational similarities.

### Deformation-Based Morphometry

The relative volume change at each voxel (Jacobian determinant, see Supplemental Data) was used to analyze volume differences. We measured relative volume differences between large regions of male and female brains by calculating the volume integral of the Jacobian determinant after normalization for absolute brain size. For the LH we also investigated the spatial location of volume changes using voxel-wise t tests between a group of 50 male and 50 females; t thresholds were corrected for multiple comparison. A parallel analysis using an all-male reference brain gave qualitatively similar results (Figure S11).

### Potential Connectivity

We used the potential synapse approach of Stepanyants and Chklovskii (2005) to estimate connectivity between PNs and LH neurons. This approach is probabilistic and uses smoothed density data as described above, in part to account for possible registration error.

## Supplemental Data

Document S1. Supplemental Experimental Procedures, Supplemental References, One Table, and Eleven Figures

Movie S1. Applying Registration Information to PN Tracings (1A) PNs are each imaged and traced in their own arbitrary coordinate system. After initial rigid (linear) registration, the tracings of PNs of the same class are already well-aligned. However, a significant improvement in the alignment of neurons of the same class can be observed after nonrigid warping registration. 5 PNs from each of the following 7 classes are displayed: 1, white; DA1, red; DM5, yellow; DM6, green; VA1d, cyan; VA1lm, blue; VM2, magenta. (QuickTime movies require free QuickTime Player 7 software for Mac or Windows available at http://www.apple.com/quicktime/download)

Movie S2. Applying Registration Information to PN Tracings (1B) PNs are each imaged and traced in their own arbitrary coordinate system. After initial rigid (linear) registration, the tracings of PNs of the same class are already well-aligned. However, a significant improvement in the alignment of neurons of the same class can be observed after nonrigid warping registration. 5 PNs from each of the following 7 classes are displayed: 1, white; DA1, red; DM5, yellow; DM6, green; VA1d, cyan; VA1lm, blue; VM2, magenta. (QuickTime movies require free QuickTime Player 7 software for Mac or Windows available at http://www.apple.com/quicktime/download)

Movie S3. 3D Animation of PN Axon Projections after Registration Traced PNs from a total of 10 different glomerular classes are displayed in the context of the MB calyx (magenta outline) and the LH (cyan outline). Initially, 2 PN classes are displayed DA1 and VM2; while their branches are somewhat overlapping in the MB calyx, they are well separated in the LH. Next, a second pair of classes are added to the movie: DL3 neurons overlap partially with DA1 neurons, while DM5 neurons show partial overlap with VM2 neurons. Notice that this is a case of correspondence between the LH and MB projection patterns: DA1 and DL3 axons are similar in both LH and MB but different from VM2 and DM5 neurons, which also show similar patterns in both structures. The movie includes 5–8 traced neurons from the following classes: 1: red, DA1: green, DL1: blue, DL3: yellow, DM5: cyan, DM6: magenta, VA1d: white, VA1lm: black, VM2: brown, VM7: orange. The axes correspond to the coordinates of the reference brain used in this study (red is x or mediolateral axis, y is green or dorsoventral axis, z is blue or anterior-posterior axis). (QuickTime movies require free QuickTime Player 7 software for Mac or Windows available at http://www.apple.com/quicktime/download)
