# Supplementary material for: Comprehensive Maps of Drosophila Higher Olfactory Centers: Spatially Segregated Fruit and Pheromone Representation
Source: Cell. 2007 Mar 23;128(6):1187–203. doi: 10.1016/j.cell.2007.01.040 (PMC1885945; doi:10.1016/j.cell.2007.01.040)

Cell, Volume *128*

Supplemental Data

Comprehensive Maps of *Drosophila* Higher Olfactory Centers: Spatially Segregated Fruit and Pheromone Representation

Gregory S.X.E. Jefferis, Christopher J. Potter, Alexander M. Chan, Elizabeth C. Marin, Torsten Rohlfing, Calvin R. Maurer, Jr., and Liqun Luo

Supplemental Data and Analyses

Registration Accuracy

Basis of registration accuracy estimate:
We modelled the axon branch point position separately for each axis by a class-specific mean plus a common standard deviation, corresponding to the registration accuracy that we wished to estimate. We verified that this common standard deviation is indeed not significantly different between the four glomerular classes (p = 0.300, 0.745, 0.892, for the X, Y and Z axes, respectively) using Levene's test (an ANOVA of the absolute values of the residuals from the initial model, Faraway, 2002).
Anisotropy of registration accuracy estimate: In the Results section "Registration Accuracy", we estimated the accuracy to be 2.64, 1.80 and 2.81 µm in the x, y and z axes. The difference between the accuracy in the x and y axes appears to be real ($p<0.01$, 10000 sample permutation test of difference of standard deviations). The explanation may be biological: these branch points occur just as the axons leave the axon tract and enter the LH; the axon tract at this point is running roughly parallel to the x axis (mediolateral) and therefore one would expect greater variation in branch point position in the x rather than the y axis (i.e., along the tract rather than across the tract). The z axis registration accuracy is likely to be poorer than the y resolution simply because the z axis resolution of the original images is not as good as the xy resolution – as is typical for essentially all confocal microscopes.
Stereotypy of PN branch point positions: We used two approaches to demonstrate the stereotypy of axon branch positions for four different glomerular classes. The first approach used a multivariate analysis of variance (MANOVA, using the base implementation in **R**) very similar in style to the 3 independent linear models used to estimate registration accuracy above:

```
fit <- manova (XYZ ~ Glomerulus)
```

i.e. MANOVA using branch point (X, Y, Z) coordinates as the three response variables and Glomerulus (a factor with four levels) as the predictor. There was a significant difference across glomeruli (p= 1.913e-12 for the default Pillai-Bartlett statistic). Separate analyses of variances for each axis indicated that there is a significant difference by glomerulus in all three axes (p = 3.31e-06, 1.11e-05 and 1.72e-05, respectively).

We also used linear discriminant analysis (LDA) to predict the glomerular class of the 40 PNs presented in Figure 1E-F on the basis of major branch point position. The LDA training success rate was 34/40 and the test success rate, using leave one out cross-validation was 32/40. The classification success rates were not significantly across the 4 glomeruli (Chi squared test, $p = 0.65$).

The cross-validation step uses the data for 39 samples to predict the class of the 40th sample and therefore is a true test of classification accuracy. If classification were at the level of chance, the success rate would be 10/40 since there are 4 PN classes (DL1, DM2, DM6, VC2). To confirm the significance of this classification accuracy, we carried out LDA on data with randomly permuted labels, again calculating the cross-validation error. For 1000 permutations, the mean accuracy rate was 10.59/40 and never exceeded 23/40; the critical values for $\alpha=0.05$ and 0.01 were 16/40 and 18/40 respectively, confirming the significance of our original classification accuracy of 32/40.

Statistical analysis of axon bundles revealed after registration: Axons originating from different neuroblasts seem to form distinct bundles (Figure 1G). We verified this statistically by calculating the position of each axon as it intersected a plane perpendicular to the axon tract (iACT) just before it entered the mushroom body. 191 PNs from 24 glomerular classes were used as input data. Note that although these positions have 3D coordinates, they are all on one plane, so they have only 2 degrees of freedom. The plane cutting the iACT is parallel to the Y axis, so we converted the 3D positions to 2D coordinates along the standard Y axis and the appropriate combination of the X and Z axes. The positions of axons from the three developmental groups (lateral neuroblast, embryonic, and larval born dorsal neuroblast) were significantly different along at least one of the two axes:

Larval lateral vs Embryonic dorsal: $p= 0.0075$, XZ axis
Larval dorsal vs Embryonic dorsal: $p= 0.0022$, XZ axis
Larval lateral vs Larval dorsal: $p= 0.0002$, Y axis

A mixed-effects model (Pinheiro and Bates, 2000) was used to account for the potential correlation in axon position of PNs of the same glomerular class.

There was in fact a significant association between glomerular class and axon position (MANOVA, $p< 2.2e-16$). When two separate linear models were constructed to predict iACT axon position along the XZ and Y axes using glomerular class as a predictor, both were highly significant (adjusted $R^2=0.226$ and 0.3968; $p=3.018e-06$ and $1.025e-13$, respectively). The standard deviation in the location of axons of the same glomerular class was 2.45 µm for the XZ axis and 2.53 µm for the Y axis. These values result from an identical statistical logic to the earlier registration accuracy calculations of LH branch point position, but describe the registration accuracy at a completely different brain location. It is therefore very interesting to see such similar values (~2.5µm).

Independent component analysis of PN projection patterns

In addition to the clustering approach presented in the main text, we took a second approach to characterising the spatial organisation of the LH using a form of dimension reduction. This can be cast as an attempt to find that set of high dimensional prototypes that when linearly combined best models the original high dimensional observations. Although the density data is very high dimensional, there is also a great deal of statistical structure in the data – the density data vary smoothly across space and adjacent voxels are correlated i.e. likely to have similar density values. Classical dimension reduction

techniques such as principal components analysis (PCA) do not take structure of this sort into account. A more recently adopted technique, Independent Component Analysis (Hyvärinen and Oja, 2000), can be advantageous with data of this form, because it attempts to find statistically independent components within the source data; this implicit dependence on the statistical structure of the input data often results in more spatially smooth components than PCA analysis and this was the case with our data set (data not shown).

We considered our data on PN projection patterns as a 4D data set with 3 spatial dimensions and a fourth dimension corresponding to 35 different classes of PN. We used ICA to ask whether it is possible to find canonical patterns of projection into the LH that capture the 3D spatial pattern of the major forms of input. For both the LH and MB calyx we found three independent components that captured the structure of the data; these components are plotted in Figure S6.

LH Gal4 Lines

acj6-GAL4 and NP5194-GAL4 do not label the same LH neurons. This was determined by three different methods: 1) staining of NP5194-GAL4, UAS-nucLacZ animals with anti-lacZ and anti-acj6 (DSHB, Univ. of Iowa) and anti-lacZ showed no overlapping expression; 2) the birthdates of neurons are different: heat shocking to induce mitotic recombination of acj6-GAL4 MARCM flies at 70-74 hr ALH did not give rise to any LHN clones (out of~200 brains); 3) simultaneous labelling of acj6-GAL4 and NP5194-GAL4 with UAS-CD8:GFP identifies two separable neuron fascicles.

Volumetric Dimorphism: Comparison of Different Reference Brains

Our main study used a reference brain composed of 16 co-registered brains, 14 female and 2 male. Co-registration was achieved by registering 15 of those brains to a single female seed brain and the registered images were then averaged. Our reference brain is therefore predominantly female. During the course of our study we registered many male and female brains to this reference brain. We were therefore able to look for evidence of regional sexual dimorphism in the lateral horn by carrying out a voxel-wise analysis of the deformation fields that map these male and female brains onto the reference brain. This identified two discrete regions in the lateral horn, one of which appeared to be male-enlarged, the other female-enlarged.

Although the use of a single template brain is typical in Deformation-Based Morphometry (DBM) studies (Studholme et al., 2004), it is reasonable to wonder if the use of a predominantly female reference brain might be somehow more likely to generate spurious registrations for male brains. This would only occur if there were an actual difference between male and female brains but could potentially lead to a mislocalisation of the exact site of that difference. We carried out an initial investigation of this issue by generating a new all-male reference brain derived from 13 males. We then registered a set of 103 brains to this male reference; 85 brains registered satisfactorily (42 males and 43 females). We then carried out a deformation based morphometry analysis as detailed in the Experimental Procedures section with the following modifications. In order to enable a direct comparison with our initial analysis, we registered the original reference brain with the new male reference brain. We used this to find the coordinates in the new male reference brain of the grid of lateral horn points that were used for plots in Figures 3-7. We then found the Jacobian determinants at these transformed co-ordinates and then plotted those values at the corresponding position in the original reference brain. Spatial mismatches between areas highlighted in the DBM analyses

against the 3 reference brains may therefore originate either from the DBM analysis itself or imperfect registration of the two reference brains. In fact the correspondence is qualitatively quite good and supports the original observations. However, a detailed analysis of this issue is out of the scope of the present study.

Supplemental Experimental Procedures

Fly Stocks

Labelling of single PNs was achieved using flies of the genotype: *y w hs-FLP UAS-mCD8-GFP/+ or Y; FRT^{G13} tubP-GAL80/FRT^{G13} GAL4-GH146 UAS-mCD8-GFP*. To examine distribution of presynaptic boutons, flies of genotype *UAS-synaptotagmin-HA/hs-FLP; FRT^{G13} tubP-GAL80/FRT^{G13} GAL4-GH146 UAS-mCD8-GFP* were used for single cell analysis (Jefferis et al., 2001). For acj6 MARCM analysis: *acj6-GAL4, UAS-mCD8-GFP/ yw hs-FLP, UAS-mCD8-GFP; l(2)03563 FRT^{40A} UAS-mCD8-GFP/FRT^{40A} tubP-GAL80*; animals were heat shocked at 20-24 hrs after puparium formation. For NP5194 MARCM analysis: *y w/ y w hs-FLP UAS-mCD8-GFP; NP5194-GAL4 FRT^{G13}/ FRT^{G13} tubP-GAL80;* animals were heat shocked at 70-74 hrs after larval hatching. For NP6099 MARCM analysis: *NP6099-GAL4 / y w hs-FLP UAS-mCD8-GFP; FRT^{G13} UAS-CD8GFP/ FRT^{G13} tubP-GAL80*; animals were heat shocked at 72-78 hrs after larval hatching.

Immunochemistry

Fixation, immunochemistry and imaging were carried out as described (Jefferis et al., 2001). Primary: Mouse anti-nc82-1:20 (Wagh et al., 2006), rat anti-CD8a (Caltag, Burlingame)- 1:100, Rabbit anti-HA (Abcam, ab9110) 1:1000; rotate 3-4 days at 4C. Secondary: Alexa-568 anti-mouse 1:150, Alex-488 anti-rat 1:150; CY5 anti-rabbit 1:200. Rotate 2-3 days at 4°C. Prolonged incubation with both primary and secondary antibodies was required for homogeneous staining. GABA staining: primary, Rabbit anti-GABA (1:100, Sigma) overnight at 4°C, followed by 2 hrs with secondary.

Image Acquisition and Processing

Confocal stacks of brains were acquired using a Biorad MRC 1024 confocal microscope. The use of a standard set of imaging parameters was important for successful image registration and we adopted the following: 40x oil lens (NA=1.3) with additional 1.5x zoom (60x total), 1 micron spacing in the Z axis and 512 x 512 resolution in XY (0.33 micron pixel width). Images were pre-processed with ImageJ (http://rsb.info.nih.gov/ij/) to ensure that all had the same orientation and slice order. All images were reoriented to assume the view in Figure S1B; images of the right side of the fly brain were therefore flipped horizontally. No additional image processing was carried out.

Image Registration Algorithm

Image registration strategies can be classified in a number of ways; key differences include how corresponding points in the two images are identified and compared and what family of co-ordinate transformations are used to map the images onto one another. Identifying corresponding points can either be done manually – by an operator placing a set of identified landmarks on two images – or in an automated and global fashion by analysing the distributions of intensity values of corresponding pixels in the two images. Manual landmark identification is not only prohibitively slow for large studies, but is

also prone to operator error. The algorithm that we have selected for this study was first proposed by Rueckert et al. (1999). It is intensity based (it does not require any feature extraction or manual interaction) and allows nonlinear deformations. Image similarity is quantified by the normalised mutual information measure (Studholme et al., 1999). The transformation model is a free-form deformation based on a uniform grid of control points that move independently of each other and thus deform the underlying coordinate space. Between the control points, a spatially continuous deformation field is obtained by interpolation with third-order B-splines. Compared to linear transformations, nonlinear deformations have many more parameters. The highest-resolution deformations computed for this work were defined by a grid of 35 x 35 x 19 control points, with three parameters per control point (its x, y, z coordinates). Out of the resulting 69825 parameters, we only optimised those that corresponded to control points in the image foreground, but the remaining number of free parameters was still several orders of magnitude above the case of a standard 12 degrees of freedom affine transformation. The optimisation problem to find the best transformation is complex and usually ill-posed i.e. there is no unique global optimum and a rough energy landscape with many local optima in which the registration can become stuck. Therefore, a constraint was introduced to regularise the optimisation problem; this constraint penalises situations in which the deformation field is not spatially smooth across the brain. Enforcing a smooth deformation is biologically reasonable – a sudden change in the direction or magnitude of the deformation would suggest that there was a tear in one of the brains. Further steps to improve the computational efficiency of the algorithm include the use of a multi-resolution, multi-level strategy, starting with downsampled images and a coarser deformation control point grid (i.e. a smaller number of free parameters) and then using incrementally higher resolution images and more free parameters.

Although a detailed comparative discussion of registration algorithms is beyond the scope of this paper (see %%ref?), we believe that the algorithm that we have selected is well matched to our problem domain. Firstly the core algorithm has previously been shown to perform well for atlas-based segmentation of brain regions on a related data type, bee brains (Rohlfing et al., 2004). Second, the use of Normalised Mutual Information as a similarity measure should make the algorithm more robust to global differences in staining. Third, the use of B-spline based interpolation and the smoothness constraint is more appropriate for relatively smooth large-scale transformations and for images in which individual voxels are sampled at a high spatial density but are somewhat noisy. Alternatives such as partial differential equation (PDE) methods are usually implemented with one image vector per voxel, which is more appropriate for images with lower spatial resolution and higher signal to noise at each pixel. More generally the algorithm that we have used has the clear benefit that the spatial scale of the image data and warping can be separately controlled. Although this algorithm has performed extremely well, it is certainly sensitive to staining homogeneity (see below) as would be expected for any intensity-based registration method.

Image Registration Implementation

Standardised images were transferred to an Origin 3800 128-processor shared memory supercomputer for registration; run times were typically 15-30 minutes per brain using 12 processors (MIPS R12K CPU running at 400 MHz). By the end of the study it was possible to run registrations at similar speeds on a 4 x 2.5 GHz processor Apple PowerMac G5 but throughput was still substantially greater on the supercomputer where we could run up to 10 registrations simultaneously. The registration technique is therefore now accessible to users without specialised computing resources, but benefits from such resources when handling large batches or during initial parameter search.

The registration algorithm has a number of free parameters that need to be tuned for each application. One important parameter is the weight parameter, **e**, that controls the relative importance of the regularisation constraint enforcing smoothness of the deformation field. We tested several values of this parameter in an initial batch of 40 brains before settling on a value of **e** = 0.1 that was used for the rest of the study. The adopted value was somewhat conservative; some brains would have registered better with a less restrictive value (allowing more variation in the direction and extent of deformation across the image) but in turn a few brains would have had excessive deformation, that was not anatomically plausible.

A perl script coordinated the registration of large batches of images. For each sample brain two major files were generated: one describing the deformation required to map the reference brain onto that sample and the second containing a reformatted version of the sample brain into the co-ordinate space of the reference brain. These files were transferred onto an Apple PowerMac G5 for subsequent analysis. The quality of the registration was manually assessed by comparing the reformatted stack with the reference brain using the ImageJ plugin SyncWindows. Only those sample brains that showed excellent overlap with the reference in the MB and LH regions were retained for further processing.

Registration Success

In total, 54.4% (313/575) of all brains registered well enough to be considered for subsequent analysis. The most common reason for registration failures seemed to be inhomogeneous staining with the presynaptic nc82 marker; optimisation of immunochemistry procedures (as described above) resulted in >80% success in the later stages of the project. Another important consideration is the quality of the template brain. There are several factors to consider. First the template brain must have high signal to noise. Second it must contain all the brain regions of interest and a significant margin of safety; we would recommend an extension of at least 10-20% in each axis since registration is poorer at the image boundaries. Third the template brain should not be a shape outlier. In this study we used an average template brain to improve signal to noise. Anecdotally, we would recommend this for brain subregions with slightly noisier, but spatially well-sampled images; the benefit was much less clear for whole brain images. When constructing an average we typically selected 3 or 4 candidate seed brains, registered a group of 40-50 brains to these seed brains and then scored registration success. We selected the seed brain that gave the highest registration success and generated an average brain from the subset of brains that registered very well. It is essential that these candidate template brains are of the highest quality obtainable or the generation of a high performance average template brain can be fiddly and time consuming.

Coordinate System and Anatomical Tracing

For those brains that registered adequately, we also traced the neuron in the green channel in order to have a precise geometric representation of its branching structure. The co-ordinate convention used throughout the study is that the origin (0,0,0) is at the top left corner of the foremost slice of the image stack (Figure S1B_1). This corresponds to the anterior dorso-medial corner in anatomical terms. Excursions in lateral, ventral or posterior directions from this origin are considered positive movement in the x, y and z axes respectively; this is a right-handed coordinate system. Tracing was carried out for each image stack with the origin defined in this manner.

Two procedures were used for tracing neurons, one using the software Neurolucida (MicroBrightfield, Colchester, VT) and the second using Amira (Mercury Computer Systems, Berlin). In the first case, tracing was completely manual. Later we switched to the use of a recently developed module that allows semi-automated tracing of neuronal structure (Schmitt et al., 2004; Evers et al., 2005) in Amira. In this second approach, branch points and segment end points are manually defined and an automated algorithm based on local intensity gradients fits the centre line and diameter of the intervening neurite at regular intervals; we used a 0.5 micron step size with this module and resampled the Neurolucida data to the same step size. The outlines of the LH and MB were traced in Amira for each optical section of the reference brain.

The tracing for each specimen was then transformed into the master coordinate system by applying the inverse of the deformation calculated when that brain was registered to the master reference brain. The inverse deformation is required because the forward transformation is used for reformatting the intensity images maps from the reference co-ordinate space to the specimen space, whereas the co-ordinates of the tracings are defined in the specimen space and must be transformed into the reference space. Because our nonrigid transformation model (like the vast majority of nonrigid transformation models) is not closed under inversion, the inverse deformation cannot be explicitly expressed. Instead, we used a numerical approximation procedure that uses the known forward transformation and the inverse of its local Jacobian matrix to obtain successively more accurate estimates of the inverse transformation for given specimen space co-ordinates. Since the forward transformation is known, we can compute the exact error of the approximation. While this does not guarantee convergence of the numerical inversion procedure, it allowed us to detect the rare cases where the approximation did not converge, for example due to a highly warped coordinate space. In these cases, we performed a full local search to obtain the inverse transformation. We have made the original and transformed tracings publicly available on the project website along with the tool used to carry out this transformation.

Density Maps

Data analysis was carried out in the statistical package **R** (R Development Core Team, 2005) using custom-written software to read and visualise representations of the neuronal branching structure and the structure of brain regions.

Data smoothing was carried out in R using a custom routine based on the function GaussSmoothArray in the package **AnalyzeFMRI** (R and companion packages are available at http://www.cran.r-project.org). Each neuron was divided into segments of length less than or equal to 0.5 microns. The central points of each segment were then binned onto the desired grid of evaluation voxels and a discrete Gaussian kernel of size at least +/- 2 sigma in each dimension was applied. For the LH we chose a value of sigma = 2.5 microns (kernel width 9 voxels) based on our assessment of the variability with which biologically similar points mapped together (Figure 1F). The data were evaluated at the centres of a grid of 50^3 equally spaced voxels with side 1.4 microns, covering the space (95:165, 60:130, 0:70) in the master coordinate system; note that this implies the first evaluation point is at (95.7, 60.7,0.7). Density data were normalised so that the value of each voxel represents the mean number of microns of arbour per cubic micron of brain expected for one neuron of that class at that point in space; this corresponds to the arbour or skeleton density metric used in (Stepanyants and Chklovskii, 2005; Shepherd et al., 2005) and has units of microns per cubic micron = μm^{-2}. In order to make a two-dimensional projection of this data, each column of voxels was integrated along the projection axis to give a

2D skeleton density now expressed in µm per $\mu m^{-2} = \mu m^{-1}$. For example for the z axis, this is the sum of the arbour densities at each (x,y) position multiplied by the depth of each voxel i.e. $\rho_{ij} = \sum_k \rho_{ijk} \delta z$.

For the MB the volume used was (45:115, 60:130, 60:95) i.e. 50 x 50 x 25 voxels. We had no *a priori* estimate for a sensible bandwidth for the smoothing kernel but after some experimentation (see Figure S5), we opted for σ = 3.5 µm, balancing the need for a larger kernel bandwidth because of the sparseness of MB bouton data against obvious oversmoothing; the discrete kernel was 13 voxels wide. We also restricted our analysis in the MB to those neuronal classes for which we had at least 4 specimens. In this case the density data refer to the mean number of boutons per cubic micron. 2D projections are again the integral of the 3D projection along the projection axis and therefore have units of boutons μm^{-2}. Density data for all neuronal classes is available on the project website both in R's native format and in the amiramesh format, which is suitable for visualisation in external programs.

Analysis of Spatial Organisation

For cluster analysis of MB and LH organisation we have two major choices to make: the distance metric and the clustering algorithm. Each of the 35 neuronal classes is considered to be an observation represented by a point in a 125,000 dimensional space. The distance between points is defined by a distance metric, most commonly the Euclidean distance, corresponding to the magnitude of the vector joining the two points in this high dimensional space. For our image data however, this metric did not seem wholly appropriate since similar images with different absolute magnitudes will be scored as very different and it is sensitive to outlier points. We tested three different metrics (which gave qualitatively similar results, GSXEJ data not shown) and settled on the Pearson correlation coefficient, **r**, as a simple and efficient metric with a natural interpretation in this context. The correlation coefficient is a robust summary of whole image similarity and is insensitive to intensity scaling factors. To convert **r** to a distance over the range (0,1) we used d = (1-r)/2 as the distance metric. The choice of clustering algorithm influences the kind of structure that the eventual dendrogram will emphasise: we selected Ward's algorithm, which aims to find compact, spherical clusters.

The Mantel test measured the correlation between all the corresponding entries in the upper triangle of a pair of distance matrices. We used the Pearson (**r**) or Spearman (ρ) correlation coefficients. However, since there are N(N-1)/2 entries derived from only N observations a permutation test is carried out to determine the null distribution of the correlation coefficient; we carried out 10,000 random permutations using the R package **vegan**; corrected $p<0.0001$ indicates that no permutation produced a value of **r** or ρ that exceeded the observed value.

Independent component analysis was carried out using the R package **fastICA** (Marchini et al., 2004), an implementation of the FastICA algorithm (Hyvärinen, 1999). Data volumes were masked to remove voxels with little information: the procedure was to sum the density value at each voxel across all classes of neuron and then to include only voxels whose summated density value was at least 10% of the absolute maximum of this sum across all voxels. For the LH 24% of voxels met this criterion.

Deformation-Based Morphometry

The nonrigid registration method that we have used generates a different deformation field to map points between the reference brain and each sample brain. Since distant regions of the brain transform fairly independently these deformation fields represent a potentially useful source of morphological information. For example, we can compare if male brains must be deformed in a characteristically different way from female brains to map them onto the reference brain. If the deformation fields that map the reference brain to the individual brains in two different sample groups have consistent differences between the groups at particular locations then this is evidence for morphological differences. This approach, called Deformation-Based Morphometry (**DBM**), has recently been used to study human brain structure (Ashburner et al., 1998; Csernansky et al., 1998; Rohlfing et al., 2006). The amount of deformation can be quantified by the local volume change at any particular grid point, which we evaluate by calculating the Jacobian determinant. This is a scaling factor, corresponding to the volume of the unit cube at a particular point in the reference image space after being transformed by the registration; it will be 1 when there is no relative size difference at that point and >1 when the sample brain is locally larger than the reference.

In order to compare the volume of brain regions across the sexes, we calculated the Jacobian determinant at a grid of voxel across each brain and integrated the volume changes at each voxel for each individual brain. Each image contained about ¼ of the volume of each brain hemisphere and we found no overall difference in brain size across this whole region. We then examined differences in the volume of individual structures within the brain. We first removed any global difference in brain size by normalising the Jacobian determinant values at each point in the brain by a global scaling factor. We used the median Jacobian determinant for each brain as the global scaling factor; this is an approximation of the relative brain size compared to the reference brain. A normalised Jacobian determinant of > 1 therefore indicates that a sample brain voxel is larger than one would expect after having taken into account the overall difference in size between that sample brain and the reference brain.

The Jacobian values were log transformed before further analysis. As a scaling factor, the Jacobian values naturally have a skewed distribution with a long tail for values greater than 1. Furthermore a statistical test on log-Jacobians will give an invariant result under any smooth change of the spatial co-ordinate system (Rohlfing et al., 2006). This means that we could have transformed all of our data into a new reference brain co-ordinate system (for example an all-male reference brain) and the statistical analysis would generate identical results. Voxel-wise analysis was therefore carried out by computing the t-statistic for the log-transformed, normalised Jacobian determinants of the male and female brains at each voxel. We used 100 sample brains (50 of each sex), so there were 98 degrees of freedom for an unpaired two-sample test assuming equal variance.

Various methods can be applied to select a threshold in the face of multiple comparisons issues. The classic Bonferroni correction will be over-conservative because of correlation between values at nearby voxels. For the LH data, the Bonferroni threshold for an across-structure two-tailed alpha = 0.05 would be $t = \pm 5.09$. One approach to take into account the spatial correlation is random field theory (RFT) associated with the classic Statistical Parametric Mapping approach widely used in human brain imaging studies (Friston et al., 1995). However, the assumptions of the RFT approach are difficult to meet and would need to be carefully investigated with the transformation family implemented in our warping algorithm. We therefore

favoured an approach which is much more assumption-free, permutation testing (Nichols and Holmes, 2002).

We used the permutation approach to define a t threshold such that we had an alpha = 0.05 across the whole structure. We calculated the null distribution of the extreme values of the t-statistic across the whole LH using 10,000 random permutations of our groups of male and female brains. We then selected the 2.5% and 97.5% quantiles of this null distribution as the t threshold values indicating female-enlarged or male-enlarged regions, respectively. This procedure is inherently conservative because we are undoubtedly incorrectly rejecting many significant voxels in order to correctly reject all non-significant voxels with p=0.95.

Note that the exact regions identified by this analysis should be interpreted with some caution. The third order B-splines that are the basis functions for the warping procedure are effectively spatially filtering the “true” area of enlargement and may show warping inhomogeneities (Rohlfing, 2006). The conservative permutation test approach will also lead to a reduction in the size of the region declared significant. Both of these processes will be somewhat mitigated by the effect of averaging over many sample brains in the absence of any systematic registration biases.

Potential Connectivity

We calculated the potential connectivity between PNs and LH neurons using the approach of Stepanyants and Chklovskii (2005). We used both the direct and approximate methods described by these authors; the results were qualitatively similar. However, the number of potential synapses calculated by the direct approach depends strongly on the exact restrictions applied when an axonal and dendritic segment run more or less parallel to each other for some distance. In both calculation methods, potential synapses are defined by approaches of less than s=1µm. In the case of the approximate method a Gaussian filter with σ=2.5µm is applied to the neuronal skeletons, which are therefore converted to line densities similar to the density maps of Figure 3. This smoothing can account for biological variability and registration error and therefore gives the potential synapse number a probabilistic interpretation.

Supplemental References

Ashburner, J., Hutton, C., Frackowiak, R., Johnsrude, I., Price, C., and Friston, K. (1998). Identifying global anatomical differences: deformation-based morphometry. Hum Brain Mapp *6*, 348-357.

Couto, A., Alenius, M., and Dickson, B. J. (2005). Molecular, anatomical, and functional organization of the Drosophila olfactory system. Curr Biol *15*, 1535-1547.

Csernansky, J. G., Joshi, S., Wang, L., Haller, J. W., Gado, M., Miller, J. P., Grenander, U., and Miller, M. I. (1998). Hippocampal morphometry in schizophrenia by high dimensional brain mapping. Proc Natl Acad Sci U S A *95*, 11406-11411.

Evers, J. F., Schmitt, S., Sibila, M., and Duch, C. (2005). Progress in functional neuroanatomy: precise automatic geometric reconstruction of neuronal morphology from confocal image stacks. J Neurophysiol *93*, 2331-2342.

Faraway, J. J. (2002). Practical Regression and ANOVA using R).

Friston, K. J., Holmes, A. P., Worsley, K. J., Poline, J. B., Frith, C., and Frackowiak, R. S. J. (1995). Statistical Parametric Maps in Functional Imaging: A General Linear Approach. Human Brain Mapping *2*, 189-210.

Hallem, E. A., and Carlson, J. R. (2006). Coding of odors by a receptor repertoire. Cell *125*, 143-160.
Hyvärinen, A. (1999). Fast and Robust Fixed-Point Algorithms for Independent Component Analysis. IEEE Transactions on Neural Networks *10*, 626-634.
Hyvärinen, A., and Oja, E. (2000). Independent Component Analysis: Algorithms and Applications. Neural Networks *13*, 411-430.
Jefferis, G. S. X. E., Marin, E. C., Stocker, R. F., and Luo, L. (2001). Target neuron prespecification in the olfactory map of Drosophila. Nature *414*, 204-208.
Marchini, J. L., Heaton, C., and Ripley, B. D. (2004). fastICA: FastICA algorithms to perform ICA and Projection Pursuit.
Marin, E. C., Jefferis, G. S. X. E., Komiyama, T., Zhu, H., and Luo, L. (2002). Representation of the glomerular olfactory map in the Drosophila brain. Cell *109*, 243-255.
Marin, E. C., Watts, R. J., Tanaka, N. K., Ito, K., and Luo, L. (2005). Developmentally programmed remodeling of the Drosophila olfactory circuit. Development *132*, 725-737.
Nichols, T. E., and Holmes, A. P. (2002). Nonparametric permutation tests for functional neuroimaging: a primer with examples. Hum Brain Mapp *15*, 1-25.
Pinheiro, J. C., and Bates, D. M. (2000). Mixed-effects models in S and S-PLUS (New York, Springer-Verlag).
R Development Core Team (2005). R: A language and environment for statistical computing (Vienna, Austria).
Rohlfing, T. (2006). Transformation Model and Constraints Cause Bias in Statistics on Deformation Fields. Paper presented at: Medical Image Computing and Computer-Assisted Intervention --- MICCAI 2006: 9th International Conference, Copenhagen, Denmark, October 1--5, 2006, Proceedings (Berlin/Heidelberg, Springer-Verlag).
Rohlfing, T., Brandt, R., Menzel, R., and Maurer, C. R., Jr. (2004). Evaluation of atlas selection strategies for atlas-based image segmentation with application to confocal microscopy images of bee brains. Neuroimage *21*, 1428-1442.
Rohlfing, T., Sullivan, E. V., and Pfefferbaum, A. (2006). Deformation-based brain morphometry to track the course of alcoholism: differences between intra-subject and inter-subject analysis. Psychiatry Res *146*, 157-170.
Rueckert, D., Sonoda, L. I., Hayes, C., Hill, D. L., Leach, M. O., and Hawkes, D. J. (1999). Nonrigid registration using free-form deformations: application to breast MR images. IEEE Trans Med Imaging *18*, 712-721.
Schmitt, S., Evers, J. F., Duch, C., Scholz, M., and Obermayer, K. (2004). New methods for the computer-assisted 3-D reconstruction of neurons from confocal image stacks. Neuroimage *23*, 1283-1298.
Shepherd, G. M., Stepanyants, A., Bureau, I., Chklovskii, D., and Svoboda, K. (2005). Geometric and functional organization of cortical circuits. Nat Neurosci *8*, 782-790.
Stepanyants, A., and Chklovskii, D. B. (2005). Neurogeometry and potential synaptic connectivity. TINS *28*, 387-394.
Studholme, C., Cardenas, V., Blumenfeld, R., Schuff, N., Rosen, H. J., Miller, B., and Weiner, M. (2004). Deformation tensor morphometry of semantic dementia with quantitative validation. Neuroimage *21*, 1387-1398.
Studholme, C., Hill, D. L. G., and Hawkes, D. J. (1999). An overlap invariant entropy measure of 3D medical image alignment. Pattern Recognition *32*, 71-86.

Wagh, D. A., Rasse, T. M., Asan, E., Hofbauer, A., Schwenkert, I., Durrbeck, H., Buchner, S., Dabauvalle, M. C., Schmidt, M., Qin, G., *et al.* (2006). Bruchpilot, a protein with homology to ELKS/CAST, is required for structural integrity and function of synaptic active zones in Drosophila. Neuron *49*, 833-844.
Wong, A. M., Wang, J. W., and Axel, R. (2002). Spatial representation of the glomerular map in the *Drosophila* protocerebrum. Cell *109*, 229-241.

Web Resources

Outline of online resources available at **http://flybrain.stanford.edu**

Data

- Raw Data of Reference Brain (pic, amira) (both seed and average)
- Label field of LH and MB calyx and surfaces for these structures
- Label field of neuropil of Reference Brain
- Traces (before and after registration). Neurolucida, SWC and AmiraMesh lineset.
- MB and LH Density Data for different classes of neuron. In R format and as separate amira files.
- Registration files for all brains used in the study
- MBLH confocal images for all brains actually used in the study (Biorad pic format)
- Sample confocal images for antennal lobe of every PN class
- Confocal stacks of GABA stained ventral PNs

Programs

- ImageJ plugins (Biorad reader /writer/Amira reader/writer/IGS raw Reader)
- Binary of registration, warp and gregxform (macosx only – others on request)
- Simple GUI for registration tools (macosx only at present)
- R analysis/visualisation functions
- Amira Script to show examples of neuronal classes

Table S1 – Neuronal classes in the study

35 different classes of PN were represented among the brains that registered within acceptable parameters. The developmental origin is tabulated here. References for the characterisation of the dendritic and axonal projection patterns of these PNs are given where they have been described by our groups before this study (Jefferis et al., 2001; Marin et al., 2002; Marin et al., 2005). Birth order has also been tabulated for the adNb lineage starting at 1 for the first born embryonic PN and restarting at 21 for the first born larval PN (DL1). The 3 classes of LH neuron included in this study are also tabulated; their parental neuroblasts are not yet characterised.

	Glomerulus	N	PNType	Neuroblast	Stage	BirthOrder	GlomRef	AxonRef	Nb
1	DC1	3	iPN	ad	embryonic				embryonicadNb
2	DL5	2	iPN	ad	embryonic	5	Marin 2005	Marin 2005	embryonicadNb
3	DL6	4	iPN	ad	embryonic	8	Marin 2005	Marin 2005	embryonicadNb
4	DM3	3	iPN	ad	embryonic	6	Marin 2005	Marin 2005	embryonicadNb
5	DP1m	8	iPN	ad	embryonic	1	Marin 2005	Marin 2005	embryonicadNb
6	VA2	1	iPN	ad	embryonic	4	Marin 2005	Marin 2005	embryonicadNb
7	VA6	1	iPN	ad	embryonic	3	Marin 2005	Marin 2005	embryonicadNb
8	VA7l	4	iPN	ad	embryonic				embryonicadNb
9	VL2p+	5	iPN	ad	embryonic	2	Marin 2005	Marin 2005	embryonicadNb
10	VM3	8	iPN	ad	embryonic	7	Marin 2005	Marin 2005	embryonicadNb
11	1	10	iPN	ad	larval		Marin 2002	Marin 2002	larvaladNb
12	D	9	iPN	ad	larval	23	Jefferis 2001		larvaladNb
13	DA3	3	iPN	ad	larval				larvaladNb
14	DC2	11	iPN	ad	larval	22	Jefferis 2001		larvaladNb
15	DC3	4	iPN	ad	larval				larvaladNb
16	DL1	14	iPN	ad	larval	21	Jefferis 2001	Marin 2002	larvaladNb
17	DM6	11	iPN	ad	larval	28	Jefferis 2001	Marin 2002	larvaladNb
18	VA1d	11	iPN	ad	larval	25	Jefferis 2001	Marin 2002	larvaladNb
19	VA1lm	11	iPN	ad	larval	29	Jefferis 2001	Marin 2002	larvaladNb
20	VA3	1	iPN	ad	larval	24	Jefferis 2001		larvaladNb
21	VM2	13	iPN	ad	larval	27	Jefferis 2001	Marin 2002	larvaladNb
22	VM7	12	iPN	ad	larval	26	Jefferis 2001	Marin 2002	larvaladNb
23	DA1	11	iPN	l	larval		Marin 2002	Marin 2002	larvallNb
24	DL3	10	iPN	l	larval		Marin 2002	Marin 2002	larvallNb
25	DM1	3	iPN	l	larval				larvallNb
26	DM2	12	iPN	l	larval				larvallNb
27	DM5	9	iPN	l	larval		Marin 2002	Marin 2002	larvallNb
28	VA7m	5	iPN	l	larval				larvallNb
29	VC2	12	iPN	l	larval				larvallNb
30	VL2p	4	iPN	l	larval				larvallNb
31	VM1	1	iPN	l	larval				larvallNb
32	vDA1	7	vPN	v	larval		Marin 2002	Marin 2002	larvalvNb
33	vVA1lm	5	vPN	v	larval		Marin 2002	Marin 2002	larvalvNb
34	vVL1	4	vPN	v	larval		Marin 2002	Marin 2002	larvalvNb
35	vmulti	4	vPN	v	larval		Marin 2002	Marin 2002	larvalvNb
36	NP5194	5	LHN						Nb
37	NP6099	6	LHN						Nb
38	acj6	10	LHN						Nb

Figure S1 –Brain Regions and Coordinates for Image Registration

(**A**) Anterior and slightly dorsal view of the left half of a fly brain. Magenta is nc82 presynaptic staining and green is membrane targeted GFP driven by the GH146-Gal4 enhancer trap, which labels approximately 90 PNs, the source of all PNs in this study. The blue box represents the area routinely imaged during this study and has a width and height of 169 microns and a depth of 88 microns. The medial, dorsal, anterior corner (yellow dot) corresponds to the origin of our coordinate system with positive excursions in the Medial-Lateral (X), Dorsal-Ventral (Y) and Anterior-Posterior (Z) axes indicated by the direction of the red, green and blue arrows (see Experimental Procedures for details).

(**B**) Visualisation of the regions corresponding to our reference brain and the specific regions used for LH and MB analysis.

(**B_1**) Anterior view of a volume rendering of our reference brain stained with nc82. The reference brain was constructed by averaging 16 co-registered brains (see Experimental Procedures) and then used for all subsequent registrations. The LH is shaded in cyan.

(**B_2**) Rotating 180° around the Z axis leads to a posterior view of the reference brain. The MB calyx is now clearly visible, shaded in magenta.

(**$B_{3\text{-}4}$**) Anterior (B_3) and Posterior (B_4) views of the MB calyx and LH with two traced PNs originating from the DP1m (red) and vDA1 (yellow) glomeruli. The DP1m PN sends its axon to both MB and LH; the vDA1 PN sends its axon only to LH. Blue bounding boxes indicate the regions that will be visualised when conducting analyses on the LH and MB calyx.

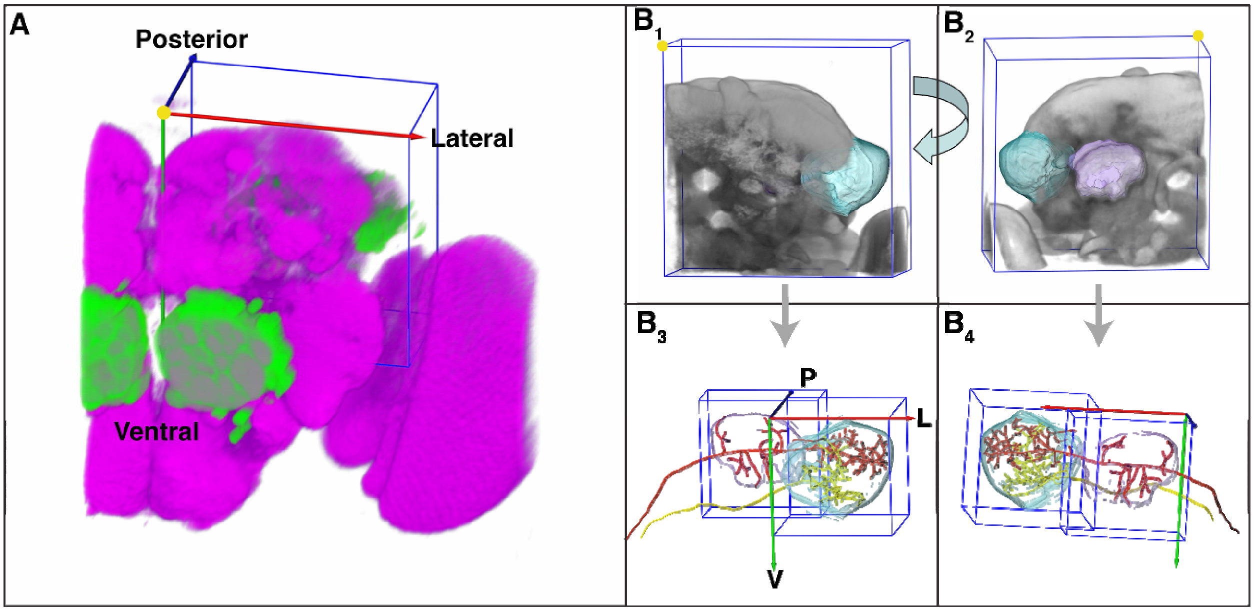

Figure S2 – Confocal Images of Previously Uncharacterised PNs

The axonal branching patterns of 13 PNs included in our main study have not previously been characterised by our group; two classes, DM1 and DM2, have previously been described by others (Wong et al., 2002). One additional novel class, VA5, is shown but no brains registered sufficiently well to be included in the main study. Panels A_0-N_0 show the position of the dendrites in the antennal lobes while panels A_1-N_1 show the axonal morphology in the LH/MB calyx. The neuroblast of origin anterodorsal (ad) or lateral (l) is indicated in parentheses. DC1 and VA7l are embryonic born PNs. Scale bars = 25µm. nc82 staining is shown in magenta.

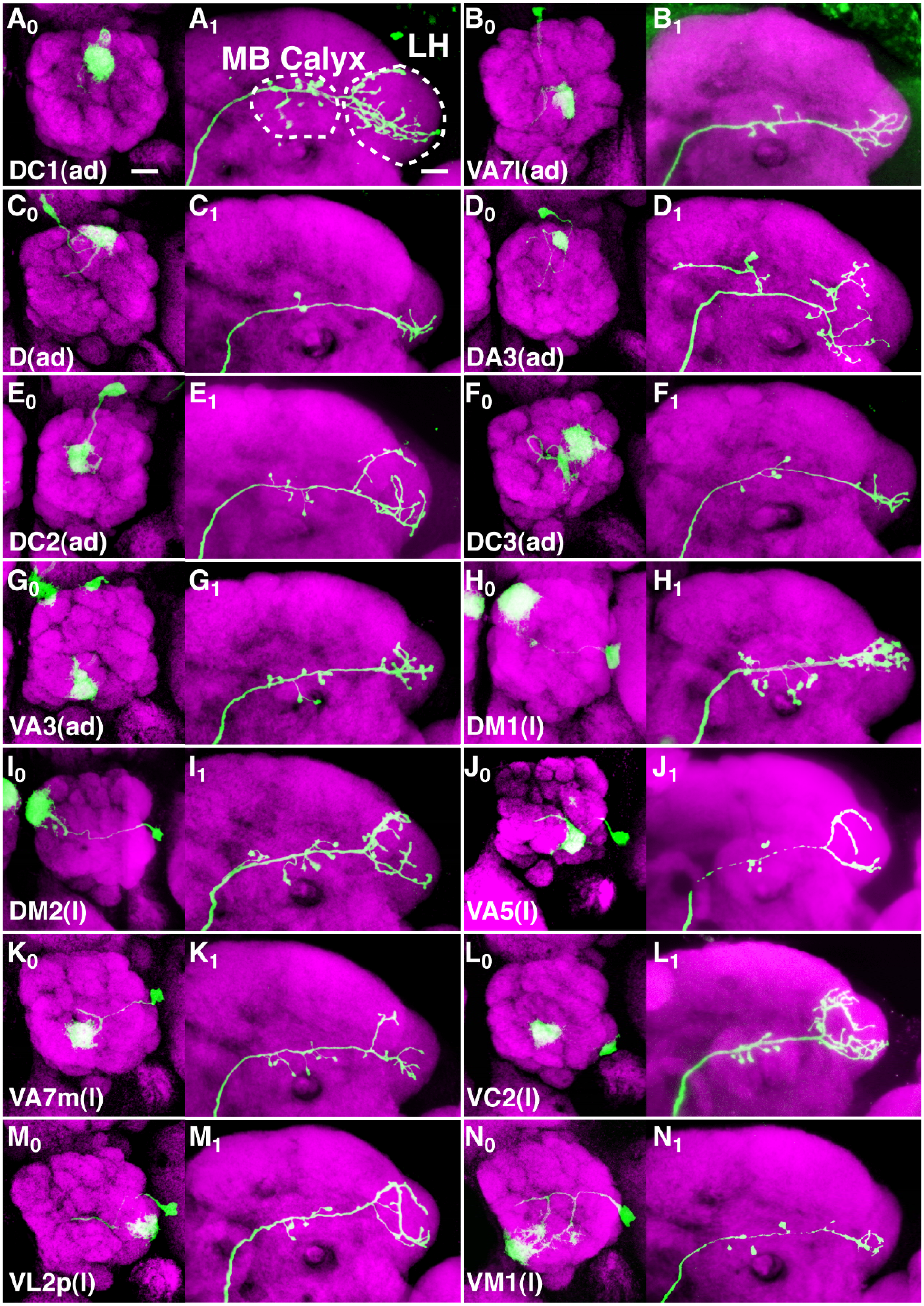
A0
DC1(ad)
A1
MB Calyx
LH
B0
VA7l(ad)
B1
C0
D(ad)
C1
D0
DA3(ad)
D1
E0
DC2(ad)
E1
F0
DC3(ad)
F1
G0
VA3(ad)
G1
H0
DM1(l)
H1
I0
DM2(l)
I1
J0
VA5(l)
J1
K0
VA7m(l)
K1
L0
VC2(l)
L1
M0
VL2p(l)
M1
N0
VM1(l)
N1

Figure S3 – Sample tracings of 35 PN Classes

Plots of single traced neurons after registration for all 35 PN classes characterised in this study. The Olfactory Receptor gene expressed in ORNs targeting each glomerulus is given in parentheses according to data from Couto et al. (2005). A question mark in parentheses indicates that the OR gene is currently unknown. PNs are coloured according to the clusters in the dendrogram in Figure 4A, which reflects the similarity of their LH projection patterns.

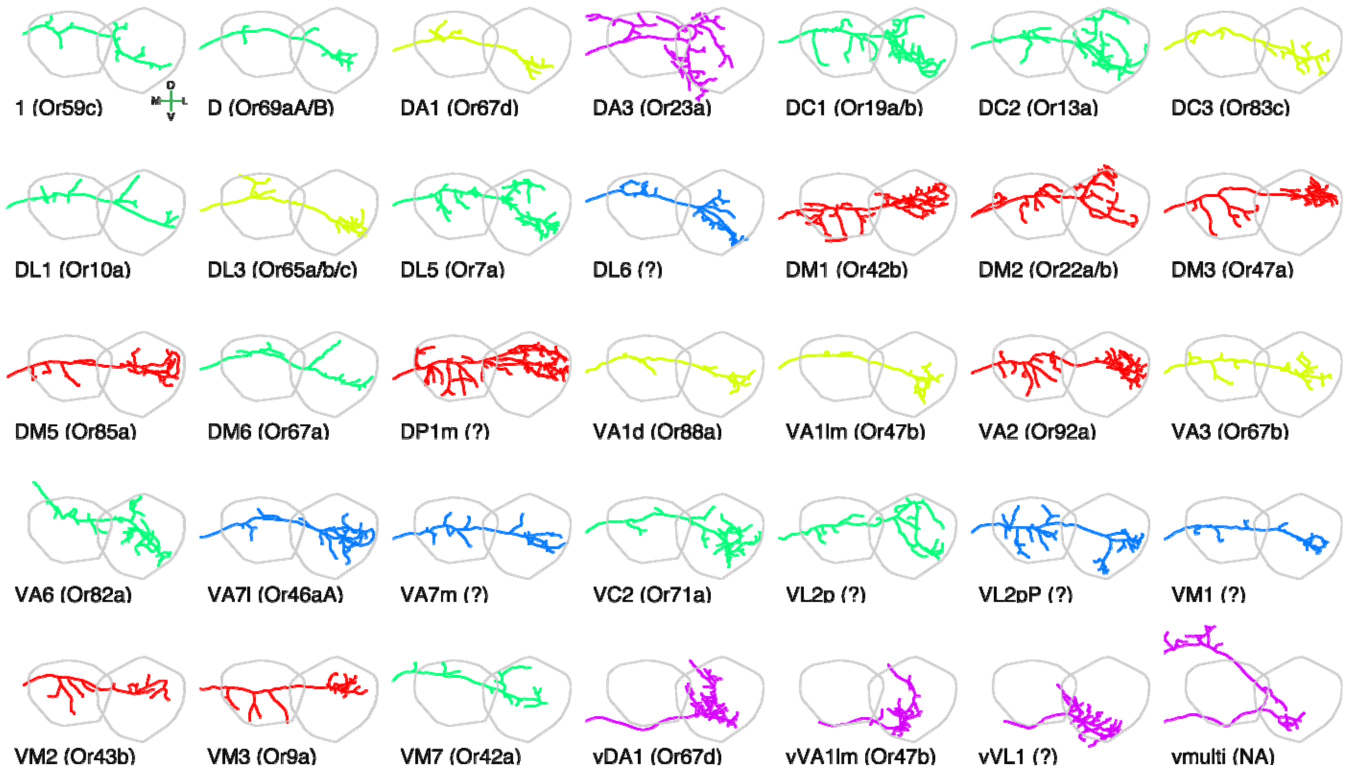

Figure S4 – Further Examples of the Distribution of Presynaptic Terminals on PNs

Localisation of presynaptic reporter UAS:synaptotagmin-HA (red) in the MB calyx (A_1-F_1) and LH (A_2-F_2) region of GH146-Gal4 (A) and five single cell PN MARCM clones as indicated (B-F). Left panels show synaptotagmin-HA; middle panel show membrane-tagged GFP (green). The merged images (right panels) show the localisation of presynaptic termini on the axonal projections (yellow). Outlines for the MB calyx and lateral region are based on nc82 staining (not shown). Scale bar, 10 µm.

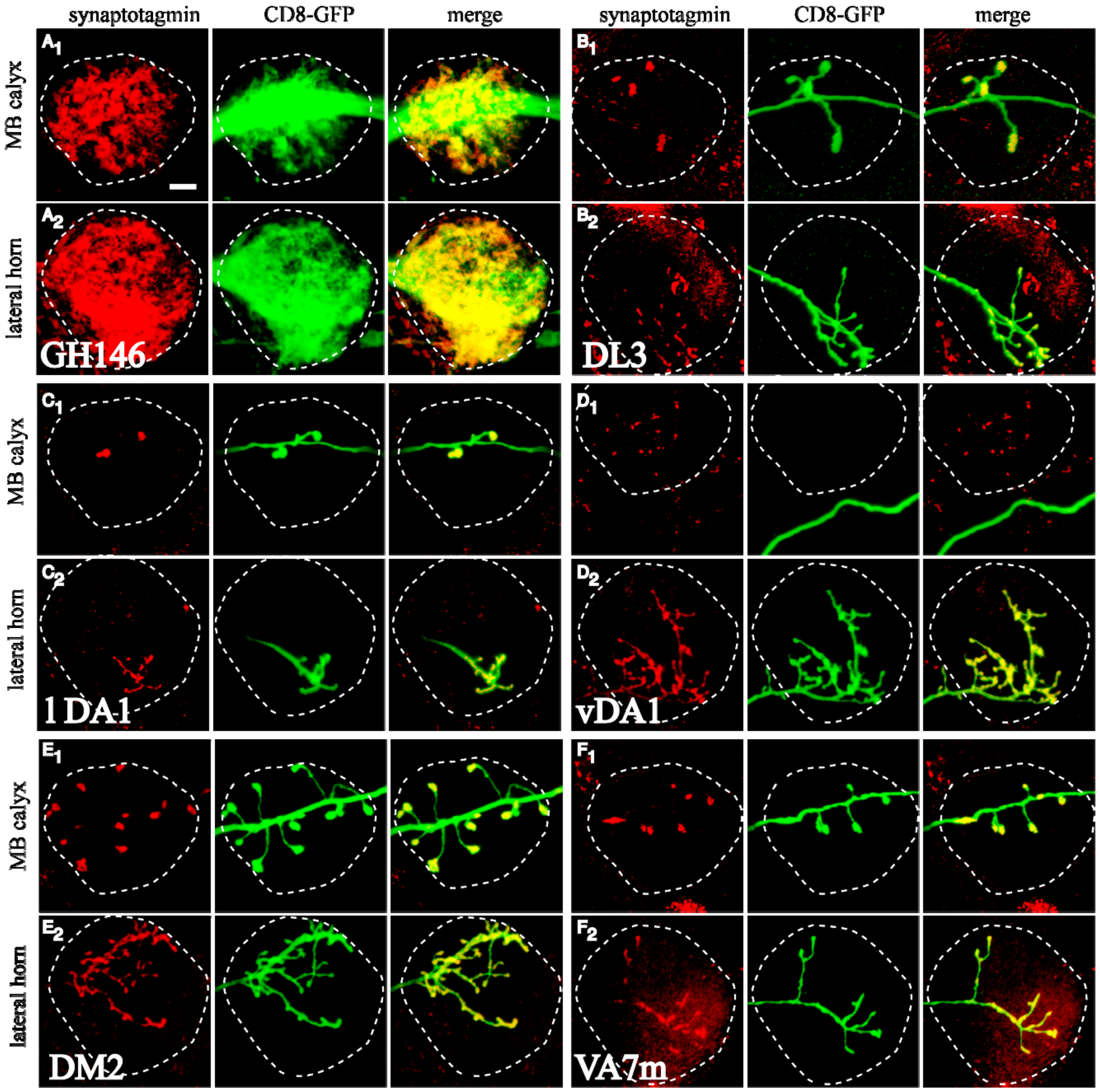

Figure S5 – Choosing the Bandwidth for Smoothing of MB Data

Anterior (XY) projections of synaptic terminal density for DM5 (n=9 neurons) and DM6 (n=11) PNs after smoothing with 3D Gaussian kernels of the indicated bandwidths. Note that there is a clear progression from under-smoothing at σ = 1.5 µm (where the individual boutons from different example neurons are distinct) to over-smoothing for σ = 5 µm. The optimal values seemed to lie in the range 2.5-3.5 µm for DM5 and 3-4 µm for DM6. The plot in the bottom right of each panel shows the kernel at the same horizontal scale as the image and is plotted over a range of +/- 10 µm around the kernel centre; the vertical scale for the kernels is arbitrary but consistent across panels.

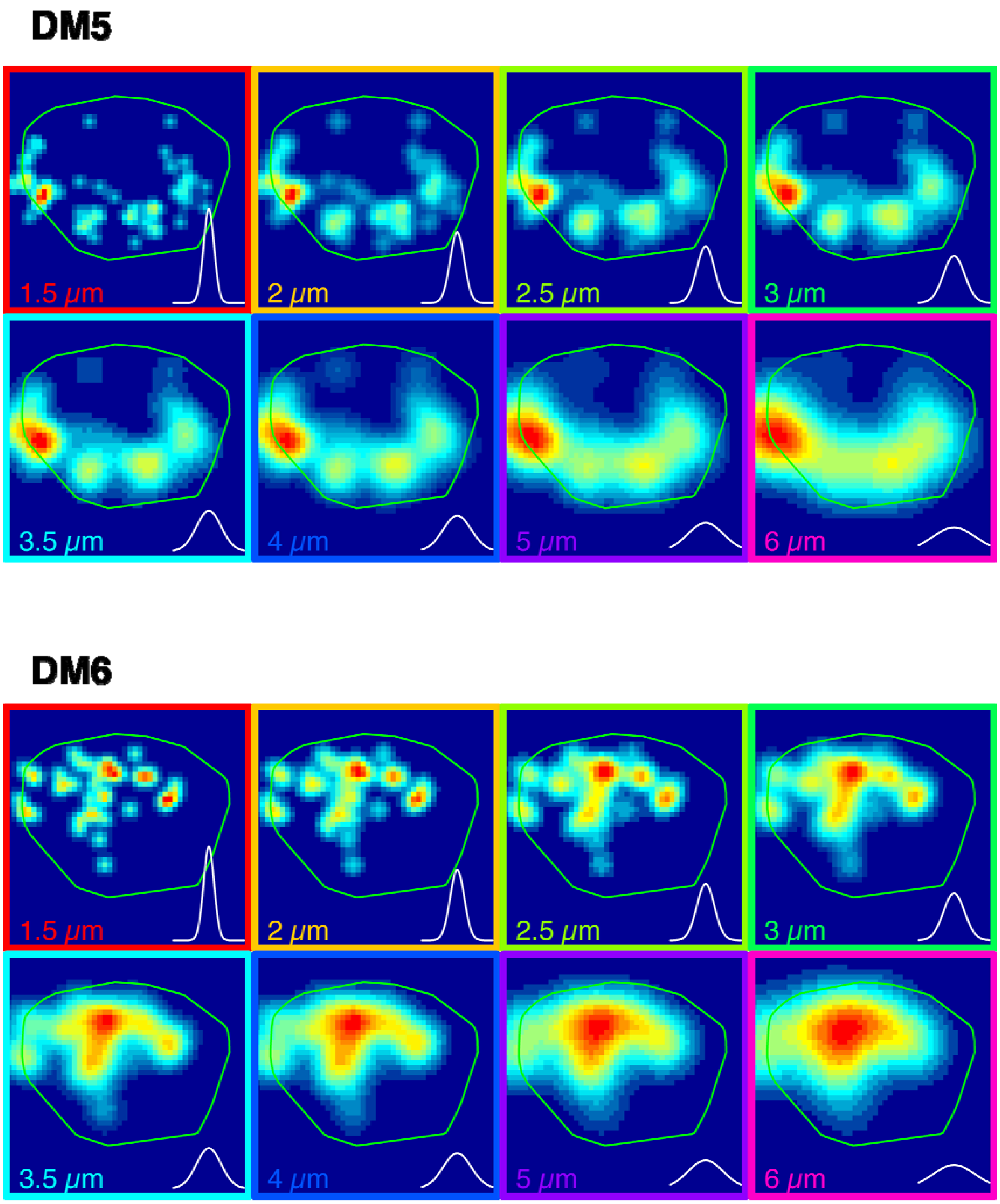

Figure S6 – Independent Components Analysis of LH and MB Density Data

(**A**) Independent components analysis of the LH synaptic density data. The LH density maps were thresholded before analysis to remove pixels that did not show a high synaptic density in at least some PN classes (see Experimental Procedures). The summed density of the three ICA components (labelled as 1-3) is plotted in anterior (XY, upper row) and dorsal (XZ, lower row) views. See Supplemental Data and Analysis for details.

(**B**) ICA analysis as in (A) for MB calyx, showing the three ICA components. The outline of the LH and MB are shown as in Figure 3; scale bars, 20 µm.

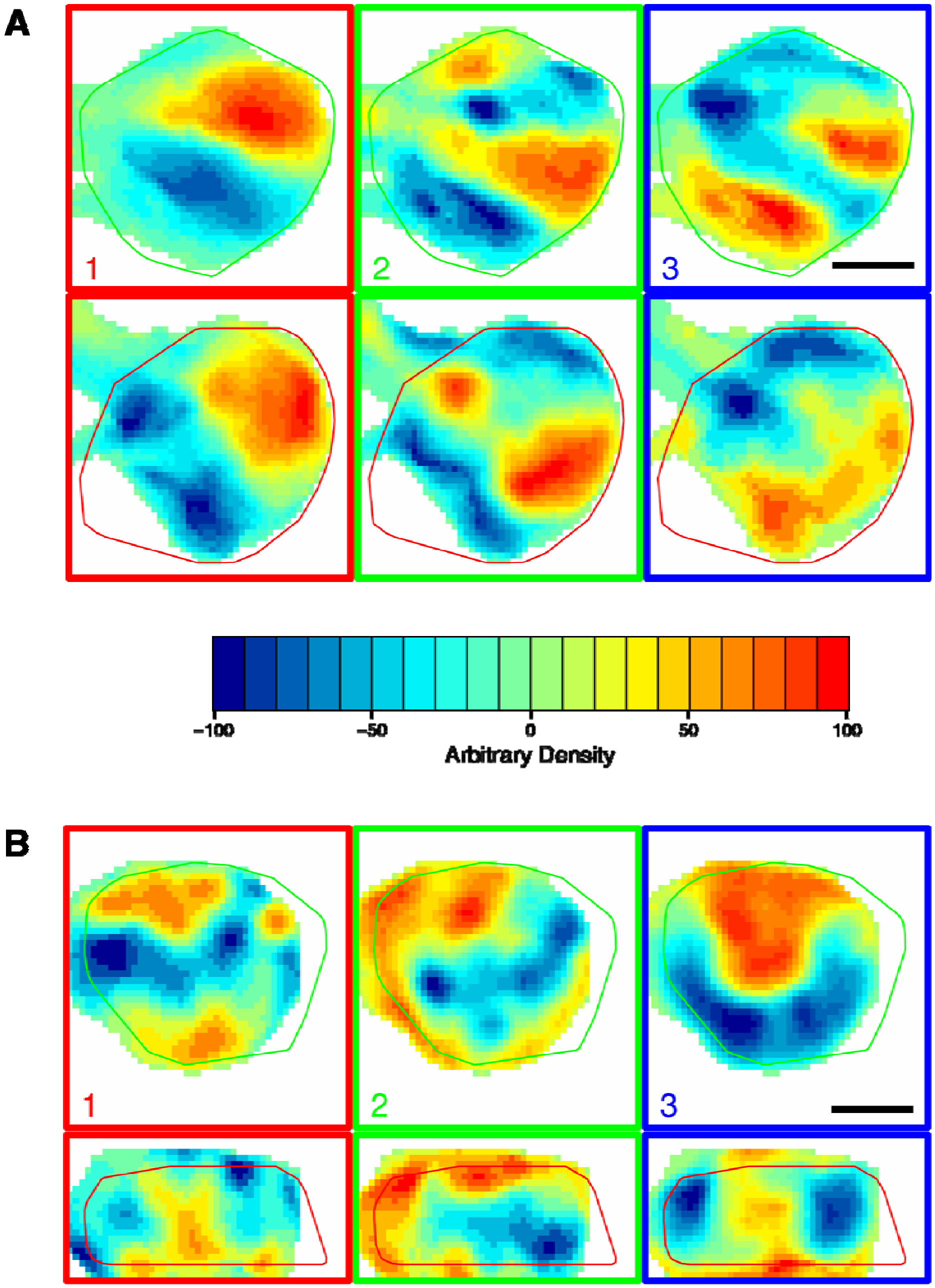

Figure S7 – Biological Correlates of MB Organisation

(**A**) The dendrogram presented in Figure 4C is re-plotted with the sensillar class of the receptor neurons that project to each glomerulus indicated to the right of the figure (coloured box).

(**B**) PNs originating from glomeruli innervated by Olfactory Receptor Neurons from different sensillar classes have somewhat distinctive spatial projections in the MB calyx. While PNs originating from antennal basiconic glomeruli (ab) can project throughout the entire calyx, trichoid (at) and palp basiconic (pb) PNs both seem to have a more restricted location in the dorso-central regions of the calyx, corresponding to clusters 2-4 but not 1 in the cluster analysis of Figure 4C-D.

(**C-D**) Predicted odour response profiles in the MB calyx. The anterior (green outline) and dorsal (red outline) activity maps of the MB calyx region are linear superpositions of the axonal density map for a particular PN class multiplied by the response rate of the corresponding odorant receptor to 3 odorants and 3 fruit extracts at 3 different dilution factors each over 4 orders of magnitude (OR data from Hallem and Carlson, 2006). The activity maps are presented in arbitrary units (heatbar) since the calculated units (μm^{-2} spikes s^{-1}) are not directly interpretable; the scales are directly comparable for all odorants and all fruit extracts. Scale bars, 20 µm.

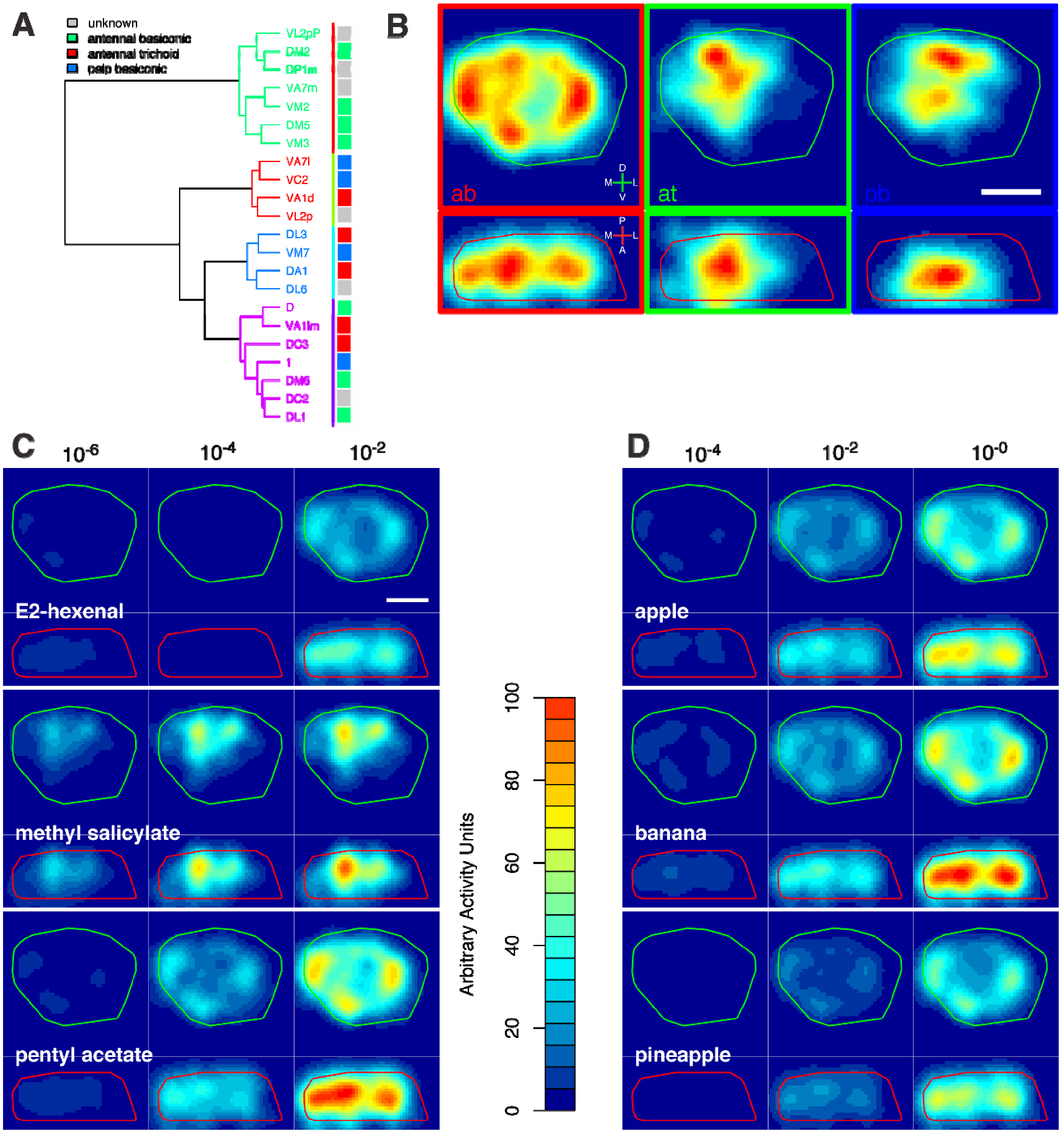
A
unknown
antennal basiconic
antennal trichoid
palp basiconic
VL2pP
DM2
DP1m
VA7m
VM2
DM5
VM3
VA7l
VC2
VA1d
VL2p
DL3
VM7
DA1
DL6
D
VA1lm
DC3
1
DM6
DC2
DL1
B
ab
at
pb
D
M
L
V
P
A
C
10^{-6}
10^{-4}
10^{-2}
E2-hexenal
methyl salicylate
pentyl acetate
D
10^{-4}
10^{-2}
10^{-0}
apple
banana
pineapple
Arbitrary Activity Units
0
20
40
60
80
100

Figure S8 – GH146 ventral projection neurons are immunoreactive for the GABA neurotransmitter.

(A) GH146-GAL4, driving UAS-mCD8GFP (green), labels identical neurons to a newly characterised enhancer trap line GH146-nucLacZ, which drives nuclear localised LacZ expression (red). The antennal lobe is outlined. A higher magnification of the yellow boxed ventral PNs in A_0 is shown in A_1.

(B) The NP3503-GAL4 enhancer trap labels 4-5 ventral PNs (green) of the vDA1 and vVA1lm classes. Co-localisation of NP3503-GAL4 with GH146-nucLacZ (red) shows that these 4-5 ventral PNs are a subset of previously characterised GH146 ventral PNs. The antennal lobe is outlined. A higher magnification of the yellow boxed ventral PNs in B_0 is shown in B_1.

(C, D) Male **(C)** and female **(D)** vVA1lm and vDA1 classes of PNs, as labeled by NP3503-GAL4 (green), are immunoreactive for GABA (red). Shown are single confocal sections. In this and all subsequent panels, the ventral PNs are outlined in solid white.

(E,F) Male **(E)** and female **(F)** GH146 MARCM single cell clones of vDA1 are immunoreactive for GABA (red). nc82 staining of the antennal lobes is shown in blue. Higher magnification of the vDA1 cell body regions is also shown in insets below, for GABA only (left) and GABA+MARCM clone (right). DAPI nuclear staining (blue) is shown in the inset for (F).

(G, H) Male **(G)** and female **(H)** GH146 MARCM single cell clones of vVA1lm are weakly immunoreactive for GABA (red). Higher magnification of the vVA1lm cell body regions is also shown in insets below, for GABA only (left) and GABA+MARCM clone (right). DAPI nuclear staining (blue) is shown in the inset for (G).
In summary, GH146+ vPNs, specifically vDA1 and vVA1lm PNs, do not always have the strongest staining for GABA, but are nevertheless GABA-positive as compared to neighbouring, GABA-negative cells. These can be appreciated much more clearly in full confocal stacks for C-H, which are available at the project website (flybrain.stanford.edu).

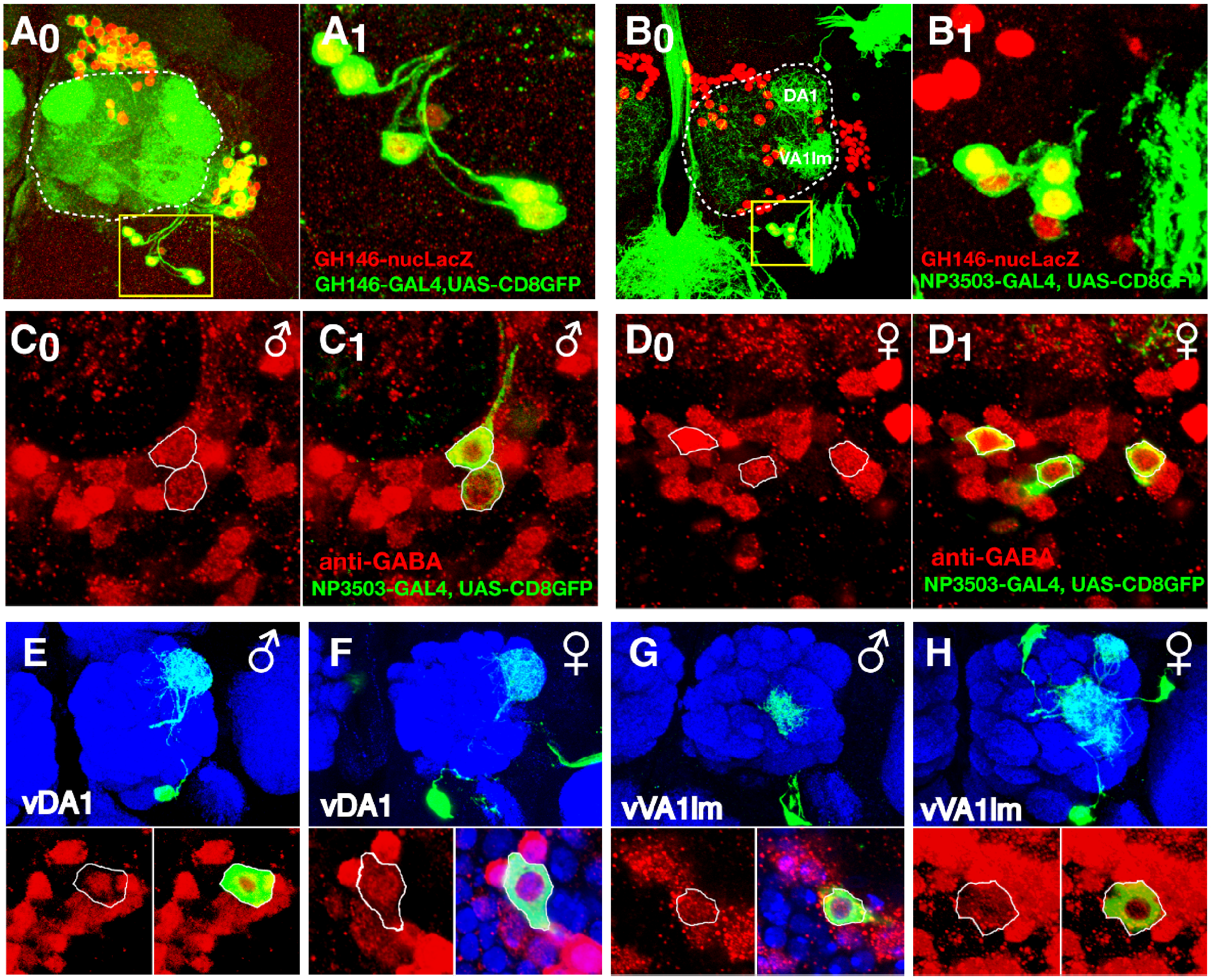
A0
A1
GH146-nucLacZ
GH146-GAL4,UAS-CD8GFP
B0
DA1
VA1lm
B1
GH146-nucLacZ
NP3503-GAL4, UAS-CD8GFP
C0
♂
C1
♂
anti-GABA
NP3503-GAL4, UAS-CD8GFP
D0
♀
D1
♀
anti-GABA
NP3503-GAL4, UAS-CD8GFP
E
♂
vDA1
F
♀
vDA1
G
♂
vVA1lm
H
♀
vVA1lm

Figure S9 – Volumetric Dimorphism in MB

(**A**) Sexually dimorphic volume of the MB calyx. Volumes are in µm^3 normalised with respect to our reference brain region, where the MB calyx occupies 25850 µm^3. The central line indicates the median, the notches represent a 95% confidence interval for the difference between medians, the box represents the 25% and 75% quartiles and the whiskers extend to +/- 1.5 times the interquartile range. The notches do not overlap, indicating a significant difference in medians.

(**B**) An anterior view of a maximum intensity z projection of an nc82 stained brain in the region of the MB calyx. The t-statistic parametric map of volume differences is superimposed on this projection. The scale bar shows the range of t-values where negative values indicate female enlarged (blue) and positive values indicate male-enlarged regions (red to green colour scale). The statistic map has been thresholded at t=2 which corresponds to approximately alpha=0.05 for a single t-test. The same anterior MB outline used throughout this manuscript is shown in light green. The multiple comparison corrected threshold of t > 3.42 (see Experimental Procedures) is indicated by black contours on the plot and by a vertical black line on the heatbar. *Scale bar, 20 µm.*

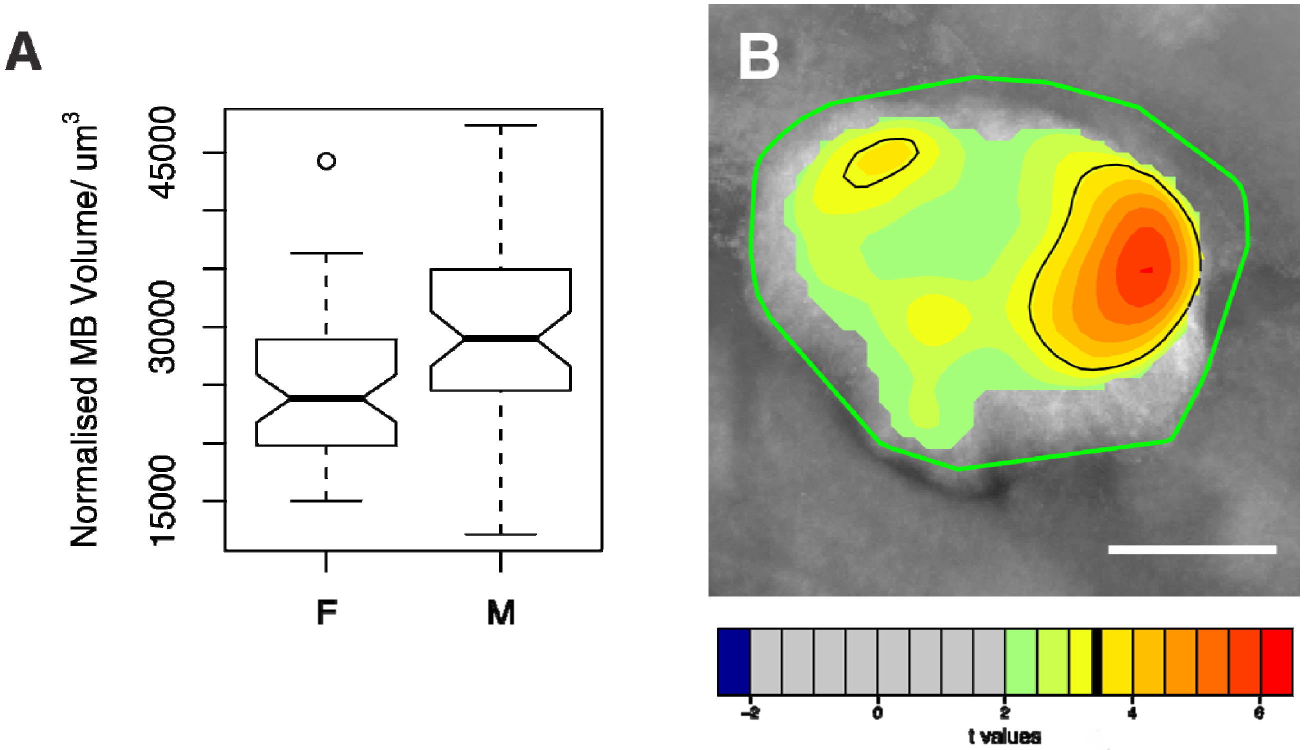

Figure S10—Single Cell Tracings of LHNs in Standard Coordinates

Anterior view of single cell tracings of 21 LH neurons plotted in the same order as the dendrogram on the side of Figure 7D and coloured according to membership of the 3 main clusters defined by potential connectivity with different PN classes. It is clear that the acj6-Gal4 line does not label a single neuronal type – differences include the extent and location of the dendritic arbours (compare acj6-4 and acj6-2). The NP5194 neurons appear to be of intermediate heterogeneity from the inspection of the individual tracings – NP5194-2 clearly has a reduced dendritic arbour compared with the other NP5194 samples, but a more extensive analysis is required to determine how many morphological types may exist.

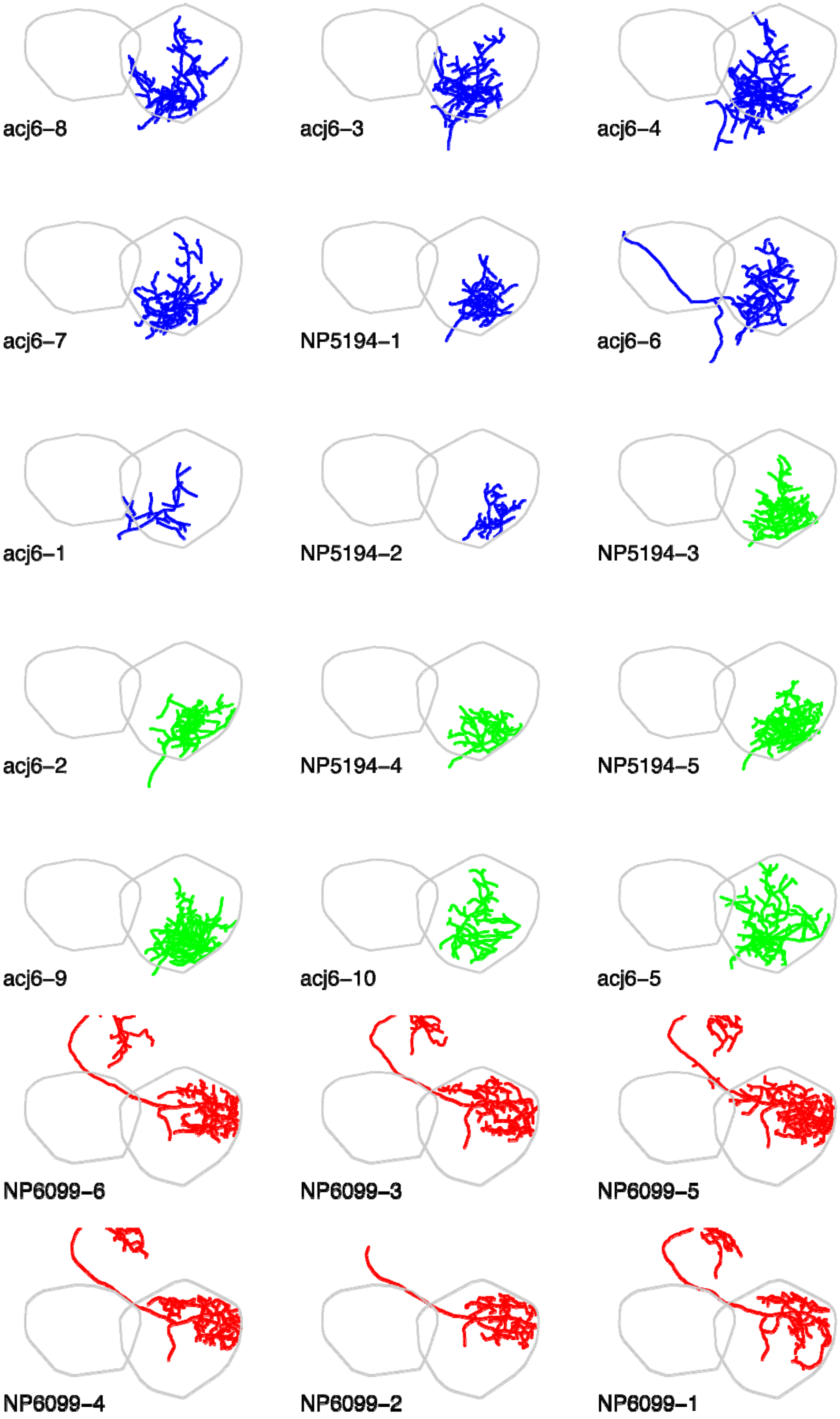
acj6–8
acj6–3
acj6–4
acj6–7
NP5194–1
acj6–6
acj6–1
NP5194–2
NP5194–3
acj6–2
NP5194–4
NP5194–5
acj6–9
acj6–10
acj6–5
NP6099–6
NP6099–3
NP6099–5
NP6099–4
NP6099–2
NP6099–1

Figure S11— Volumetric Dimorphism Using Male and Female Reference Brains

Anterior views of slices at 5 different z axis positions in the lateral horn of the t statistic parametric maps that compare the deformation fields of male and female brains. The first row uses our original largely female average brain as the reference to which all samples are registered. The second row uses a slightly modified reference brain consisting of 14 female brains. Finally the third row uses a new all male reference brain. The outline of the two regions identified by our initial analysis as male-enlarged or female-enlarged are outlined in dotted or solid white lines, respectively. The patterns of dimorphism are broadly similar. However, the t statistic signals are weaker for two reasons: 1) Fewer brains, (42 rather than 50) could be used. 2) Registration to the new all male reference brain also appears to be generally poorer than against our original reference. This was equally true for male and female sample brains (GSXEJ, data not shown).

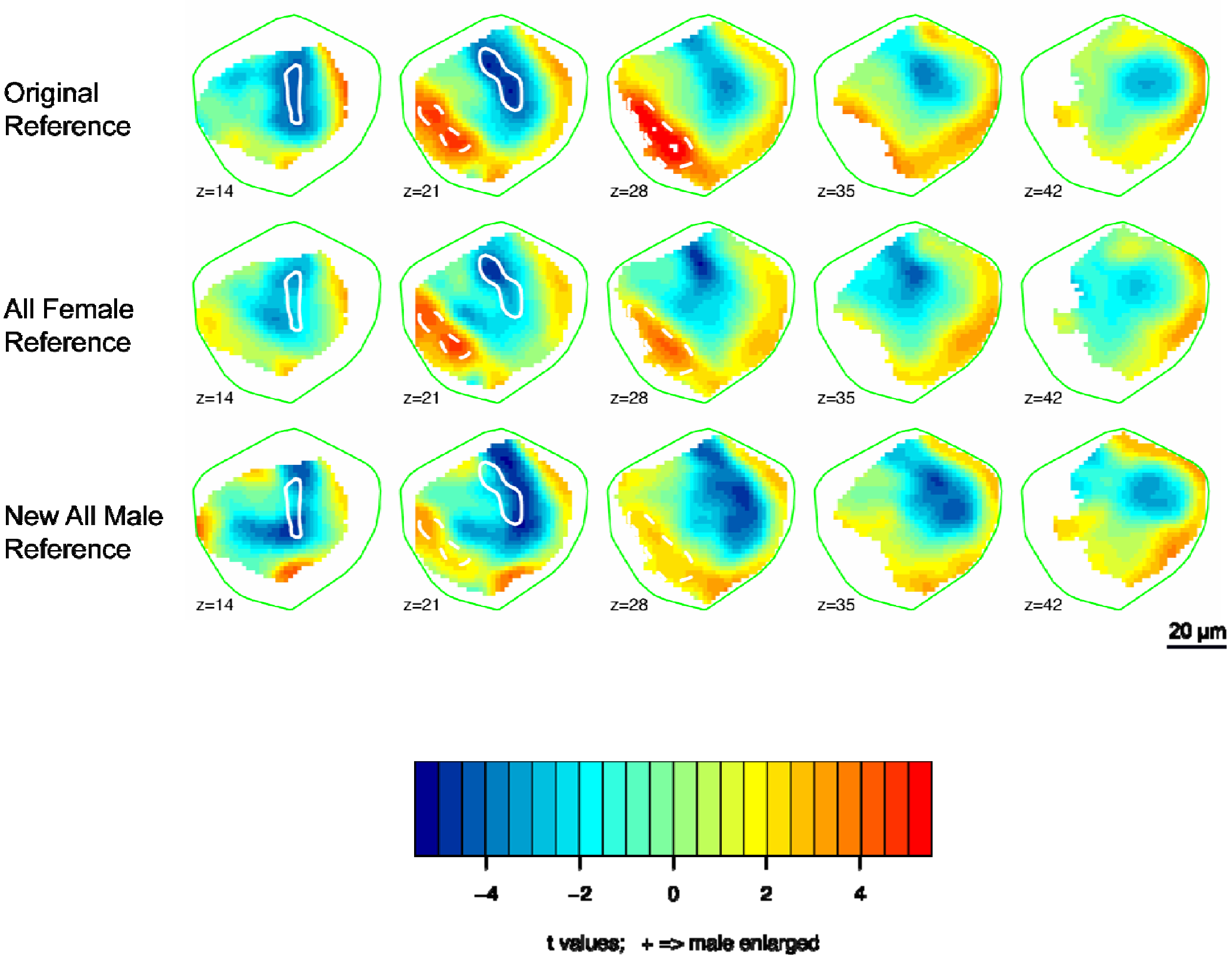

Supplement: Document S1. Supplemental Experimental Procedures, Supplemental References, One Table, and Eleven Figures [file mmc1.pdf]
